# Suppression of BCL6 function by HDAC inhibitor mediated acetylation and chromatin modification enhances BET inhibitor effects in B-cell lymphoma cells

**DOI:** 10.1038/s41598-019-52714-4

**Published:** 2019-11-11

**Authors:** María G. Cortiguera, Lorena García-Gaipo, Simon D. Wagner, Javier León, Ana Batlle-López, M. Dolores Delgado

**Affiliations:** 1 Instituto de Biomedicina y Biotecnología de Cantabria (IBBTEC) CSIC-Universidad de Cantabria, and Department of Biología Molecular, Universidad de Cantabria, Santander, Spain; 2 Servicio de Hematologia, Hospital Marqués de Valdecilla-IDIVAL, Santander, Spain; 3 Leicester Cancer Research Centre and Ernest and Helen Scott Haematological Research Unit, University of Leicester, Leicester, LE1 7HB UK

**Keywords:** Haematological cancer, Lymphoma

## Abstract

Multiple genetic aberrations in the regulation of BCL6, including in acetyltransferase genes, occur in clinically aggressive B-cell lymphomas and lead to higher expression levels and activity of this transcriptional repressor. BCL6 is, therefore, an attractive target for therapy in aggressive lymphomas. In this study romidepsin, a potent histone deacetylase inhibitor (HDACi), induced apoptosis and cell cycle arrest in Burkitt and diffuse large B-cell lymphoma cell lines, which are model cells for studying the mechanism of action of BCL6. Romidepsin caused BCL6 acetylation at early timepoints inhibiting its function, while at later timepoints BCL6 expression was reduced and target gene expression increased due to chromatin modification. MYC contributes to poor prognosis in aggressive lymphoma. MYC function is reduced by inhibition of chromatin readers of the bromodomain and extra-terminal repeat (BET) family, which includes BRD4. The novel combination of romidepsin and JQ1, a BRD4 inhibitor was investigated and showed synergy. Collectively we suggest that the combination of HDACi and BRD4i should be pursued in further pre-clinical testing.

## Introduction

Aggressive lymphomas such as diffuse large B-cell lymphoma (DLBCL) and Burkitt lymphoma (BL) are a heterogeneous group of disorders in terms of clinical behavior, biological characteristics and response to treatments^1^. Despite improvements in diagnosis and treatment, non-Hodgkins-lymphomas are still an important cause of morbidity and mortality worldwide. Combination of rituximab with anthracycline-based chemotherapy regimen, such as R-CHOP (Rituximab-cyclophosphamide, doxorubicin, vincristine and prednisone) is effective^2^ but overall >40% of patients are not cured of their disease. Thus, it is important to identify new approaches to therapy. Aggressive lymphomas are often derived from germinal center B-cells. Germinal centers are dynamic structures within normal lymph nodes, where B-cells proliferate intensely and undergo somatic hypermutation^3^, a process involving the production of DNA breaks that is essential for the formation of high affinity antibodies. Conditions within the germinal center are therefore believed to predispose to the formation of lymphomas^3^. There is a wealth of genetic evidence that BCL6 contributes to the survival of DLBCL and clinical evidence suggests that a proportion of BCL6 expressing DLBCL patients have poor clinical outcomes. The role of BCL6 in Burkitt lymphoma has not been investigated but it is expressed in all cases and is likely to contribute to proliferation and survival.

BCL6 is a master transcription factor that is essential for normal germinal center formation^4,5^. Enforced expression of BCL6 in mice is sufficient for the development of lymphomas^6^. Multiple genetic abnormalities leading to increased BCL6 expression have been described^7,8^ and BCL6 is also involved in chromosomal translocations in ~25% of DLBCL^9^. Thus, modulation of *BCL6* expression could be a potential target for therapy in lymphomas. Indeed, BCL6 inhibition using specific inhibitors was able to produce apoptosis and cell cycle arrest of these cells^10,11^ suggesting that BCL6 may be a promising therapeutic target in lymphoma^12,13^.

We and others, have recently shown that epigenetic mechanisms are involved in *BCL6* regulation^14–16^. Histone deacetylase inhibitors (HDACi) are a novel class of antitumor agents that have shown very promising results for the treatment of a number of hematologic malignancies^17,18^. Regulation of the reversible acetylation status of an increasing number of non-histone proteins, many of them being proto-oncogenes, allows to modulate a number of essential cellular processes such as protein interactions, protein stability, apoptosis, cell proliferation and cell survival^19^. Particularly, HDAC inhibitors have been shown to inhibit BCL6 function by inducing its acetylation, which leads to de-repression of its target genes^20^. Romidepsin is an HDACi with high inhibitory activity for class I histone deacetylases that is approved by the FDA for the treatment of cutaneous T-cell lymphoma or refractory/relapsed peripheral T-cell lymphoma^21,22^. HDACi synergize with other agents including hypomethylating agents in pre-clinical models of DLBCL^23^.

MYC translocations occur in 10–15% of DLBCL^1^. High expression of MYC, independent of the presence of chromosomal translocations involving MYC, is associated with poor clinical outcome in B-cell lymphoma^24,25^. There is interest in the bromodomain and extra-terminal (BET) family member BRD4, which recognizes acetylated histones and plays an essential role in the regulation of *MYC* expression^26^. BRD4 (bromodomain-containing protein-4) inhibitors^27^ such as JQ1 are able to cause *MYC* oncogene downregulation in a variety of human cancers, including leukemia and lymphoma^28^. BET inhibitors are currently being used in clinical trials^29^.

Promising data on combining HDACi with BRD4 inhibitors has been reported^18^. This combination has a specific rationale in DLBCL and BL as it potentially targets MYC in poor prognosis disease. Thus, the aim of this study was to investigate the effects of romidepsin alone or in combination with the BRD4 inhibitor, JQ1, in the treatment of aggressive lymphomas, and to identify the molecular mechanisms involved in its effects.

## Results

### Romidepsin promotes apoptosis in cells from agressive lymphomas

**Figure 1:**
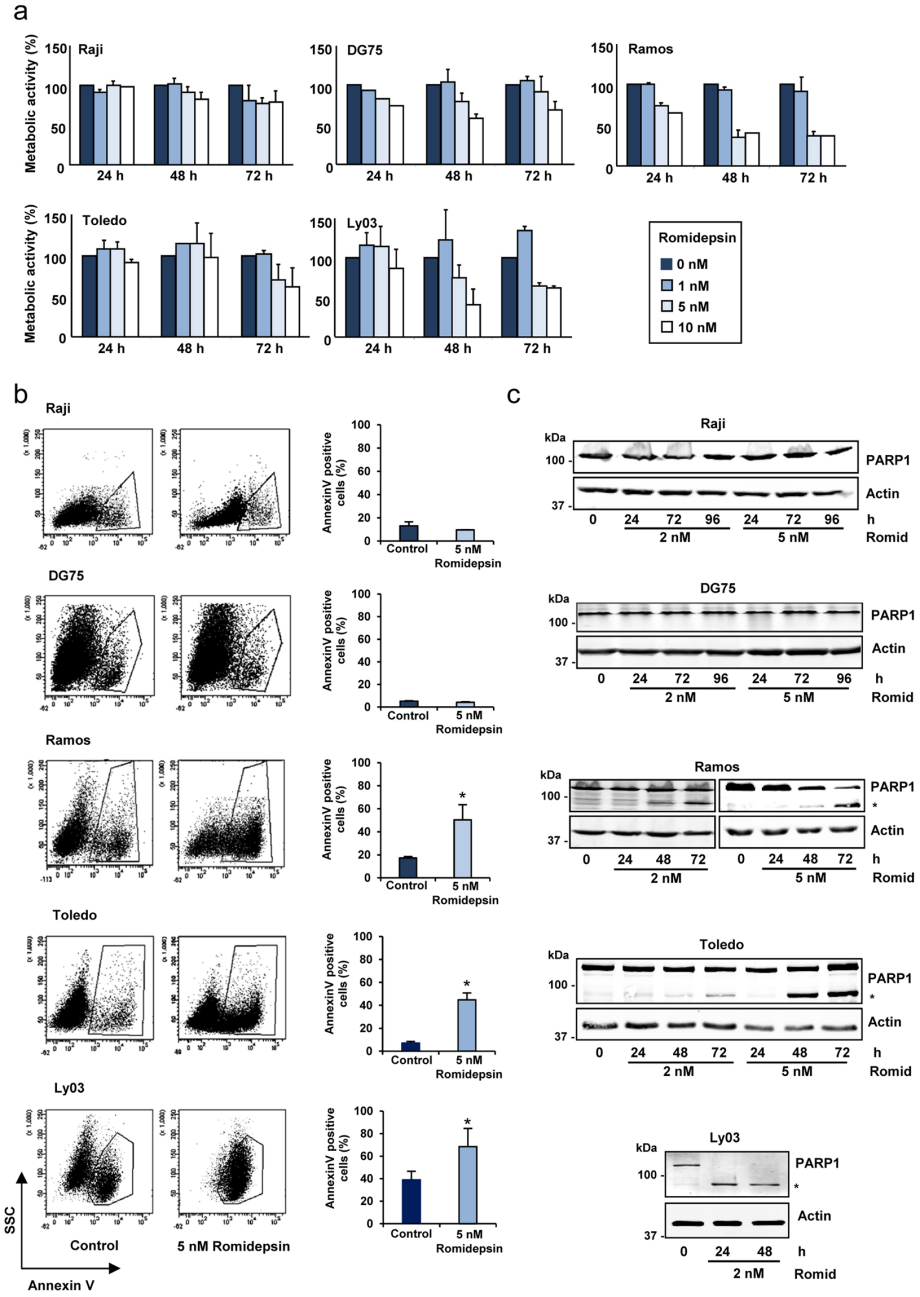
Romidepsin effect on B-cell lymphoma cells proliferation and apoptosis. (**a**) The indicated cell lines were treated with different concentrations of romidepsin and metabolic activity was determined using WST-1 method at the designated times. Untreated cells represented 100% of metabolic activity. The data show the means ± s.e.m. of four measurements in two independent experiments. (**b**) Annexin V staining to assess early apoptosis in B-cell lymphoma cells untreated (control) or cells treated with 5 nM romidepsin for 48 h. One representative experiment is shown for each cell line. The graphs on the right represent percentages of Annexin V positive cells. The data show the means ± s.e.m. of two or three independent experiments; significance difference (*p < 0.05) from the control untreated cells. (**c**) Western blot showing PARP1 and cleaved-PARP1 (indicated with an asterisk) in B-cell lymphoma cells treated with romidepsin at the indicated times and concentrations. Actin was used as loading control. The blots were cropped for improved clarity and the full-length blots were included in the Supplementary Information file.

As a first approach, we measured cell proliferation (based on metabolic activity) upon romidepsin treatment to establish a dose-response assessment and to analyze the effect of the HDACi on proliferation at different time points (Fig. 1a). Romidepsin was tested in different types of aggressive B-cell lymphoma cell lines: three Burkitt lymphoma cell lines (Raji, DG75 and Ramos), one GC-DLBCL (Toledo) and one ABC-DLBCL (Ly03) (see Supplementary Table S1).

At 48 h, Raji and DG75 cells showed little (10–20%) reduction of metabolic activity (Fig. 1a), even with the highest doses tested (10 nM). Ramos cells were the most sensitive, showing a metabolic reduction 50% after treatment with romidepsin (5 nM) while both Toledo and Ly03, showed intermediate sensitivity. Very high doses of romidepsin inhibit almost completely the proliferation of all the lymphoma cell lines studied (not shown). Given that with 1 nM concentration did not show any significant effect on the studied cell lines and 10 nM treatment resulted in cell death for the most sensitive cell lines, we chose 2 nM and 5 nM as optimal concentrations for further experiments.

To evaluate the effects of romidepsin on apoptosis, Annexin V binding was determined (Fig. 1b). No significant cell death was observed for the metabolically less-sensitive cell lines Raji and DG75, while the sensitive cell lines Ramos, Toledo and Ly03, showed significant apoptosis induction after treatment with romidepsin (5 nM) (Fig. 1b). The apoptotic effects of this drug were verified using the PARP1 cleavage assay. Cleaved PARP1 was found in the high and moderate sensitive cell lines (Ramos, Toledo and Ly03) but not in the less sensitive ones (Raji and DG75) (Fig. 1c).

**Figure 2:**
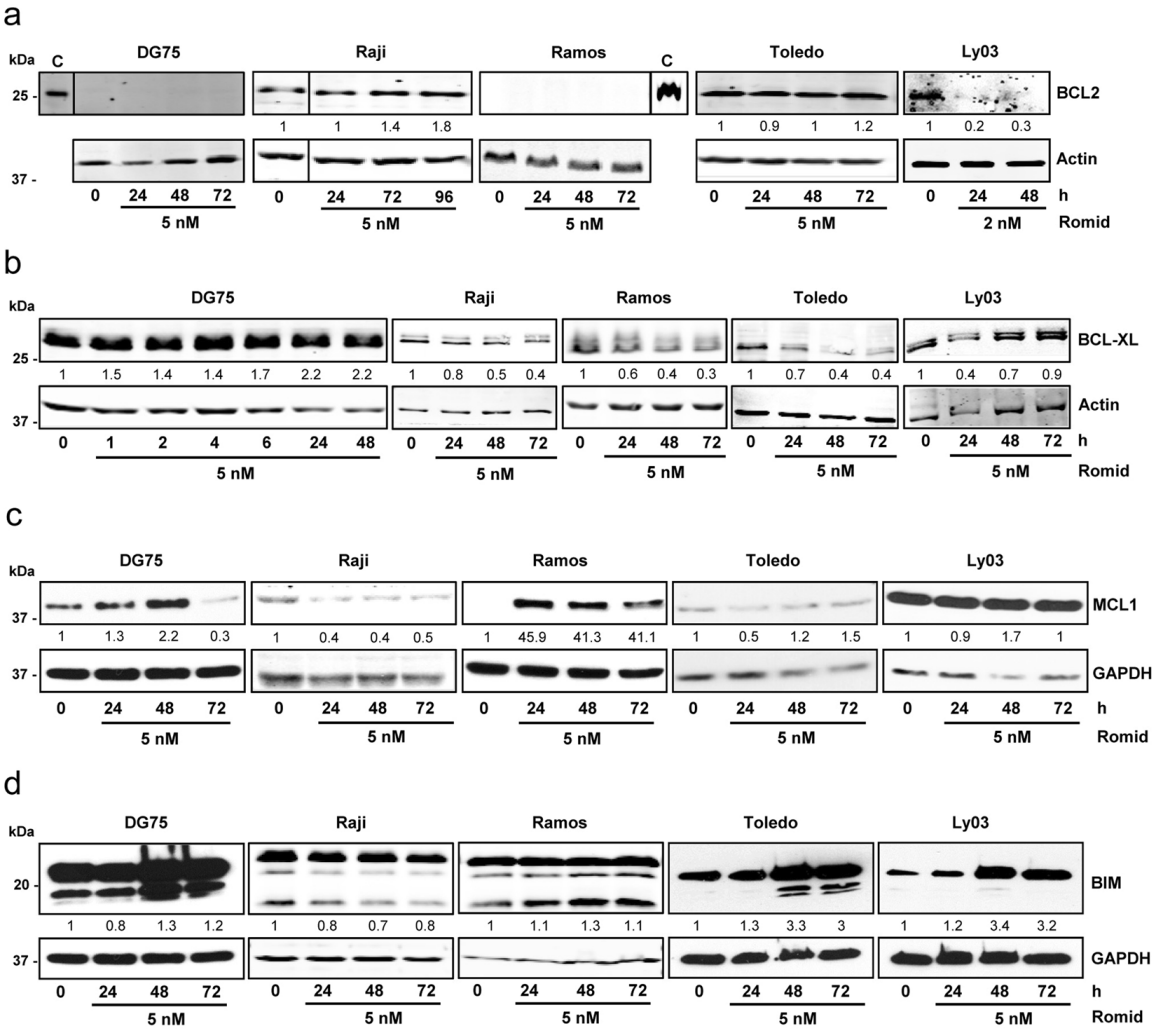
BCL2 family protein expression upon romidepsin treatment. Western blot showing BCL2 (**a**), BCL-xL (**b**), MCL1 (**c**) and BIM (**d**) protein expression in B-cell lymphoma cells treated with 2 or 5 nM romidepsin for the indicated times. Actin or GAPDH were used as loading controls. Densitometry values are shown at the bottom, normalized to the control. The blots were cropped for improved clarity and the full-length blots were included in the Supplementary Information file.

We next examined the effect of romidepsin on expression of some BCL2 family members (Fig. 2). The intrinsic apoptotic pathway can be primarily activated by chemotherapeutic drugs and various studies support its role in HDACi mediated cell death^30^. In agreement with previous reports, BCL2 expression was neither observed in Ramos or DG75 in basal conditions^31,32^ or upon treatment with romidepsin. There was little change in the level of BCL2 in Raji and Toledo while there was suppression in Ly03 (Fig. 2a), confirming previous results indicating that romidepsin is able to induce apoptosis despite BCL2 expression^33^. Other pro-survival proteins were investigated: BCL-xL levels were high in basal conditions and were reduced upon treatment in the sensitive cell lines with the exception of Ly03 (Fig. 2b), suggesting that downregulation of BCL-xL might be mediating romidepsin apoptotic effect on germinal center derived cells. Transient expression or little change was observed in MCL1 levels with romidepsin (Fig. 2c). Interestingly, the pro-apoptotic protein BIM (Fig. 2d), showed some increase with romidepsin in DLBCL cell lines (Toledo and Ly03), which was in agreement with the PARP1 cleavage pattern detected with romidepsin treatment.

Altogether, these results demonstrate that romidepsin can repress metabolic activity and induce variable levels of apoptosis associated with changes in expression of BCL2 family proteins that might be different upon the different subtypes of lymphomas.

### Romidepsin induces cell cycle arrest accompanied with p21 and p27 up-regulation

**Figure 3:**
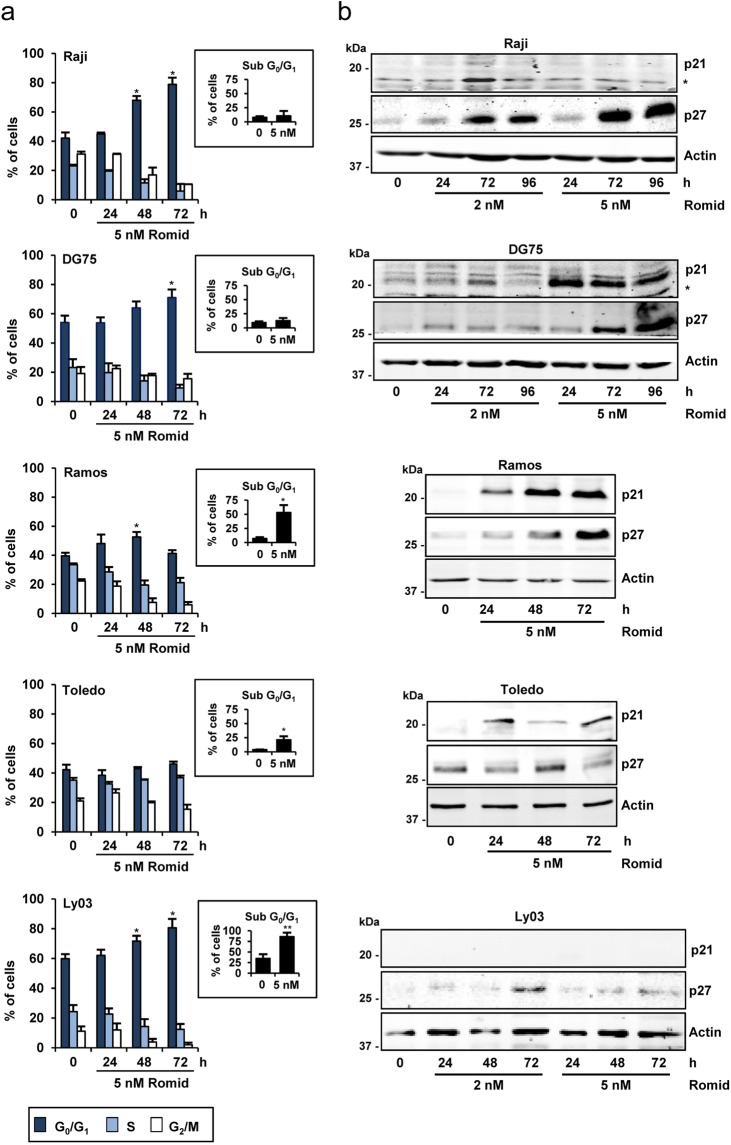
Cell cycle distribution and cell cycle inhibitors expression in B-cell lymphoma cells in the presence of romidepsin. (**a**) Cell cycle assays were performed using propidium iodide staining and flow cytometry in the indicated cell lines. Cells were treated with 5 nM of romidepsin for the indicated times. In the main graph, the fraction of cells in each phase of the cell cycle was calculated as a percentage from the total viable population. Subdiploid cells were gated out and not included in this analysis. The data show the means ± s.e.m. of two to four independent experiments; significance difference (*p < 0.05) from the control untreated cells. The inset show the fraction of cells in the sub-G_0_/G_1_ phase, indicative of cell death. (**b**) Western blot showing p21 and p27 protein expression in B-cell lymphoma cells treated with 2 or 5 nM romidepsin for the indicated times. Actin was used as loading control. Unspecific band is indicated (*). The blots were cropped for improved clarity and the full-length blots were included in the Supplementary Information file.

Romidepsin has previously been shown to induce cell cycle arrest in cells from different tissues^22^. Based on this information, we aimed to analyze the cell cycle in cell lines with different responses to the HDACi (Fig. 3a). The fraction of cells in the sub-G_0_/G_1_ phase upon 48 h of romidepsin treatment, corresponding to subdiploid dead cells (inset in Fig. 3a), is in agreement with the apoptosis observed in the sensitive Ramos, Toledo and Ly03 cells (Fig. 1). In Raji and DG75, the percentage of cells in sub-G_0_/G_1_ did not change upon romidepsin (inset in Fig. 3a), however a significant accumulation of cells in G_0_/G_1_ was found (main graph in Fig. 3a) indicating that even in those less-sensitive cells, a certain antiproliferative effect was observed. For Ramos and Ly03 the accumulation in G_0_/G_1_ was smaller but still statistically significant. The cell population distribution in Toledo cell line was not substantially affected (main graph in Fig. 3a).

Given the effects of HDACi on cell cycle arrest, we wonder whether the cell cycle inhibitors p27^Kip1^ (p27) and p21^Cip1^ (p21) were involved as has been reported for other HDACi^34^. We observed an increase of p21 protein levels in DG75 and Ramos cells treated with romidepsin (Fig. 3b). The increase in p21 in Toledo cells was no so prominent, probably reflecting the lack of cell cycle arrest in these cells. Additionally, levels of p27 were increased in Raji, DG75, Ramos and Ly03 in the presence of romidepsin (Fig. 3b). There is, therefore, no clear agreement between protein levels of p21 and p27 and the clear block in G_1_ phase that the drug provokes across all these cell lines (Fig. 3a). Overall, romidepsin induces cell cycle arrest together with increase in p21 and p27 expression in Ramos, the most sensitive cell line.

### Romidepsin provokes BCL6 downregulation and triggers plasma cell differentiation

**Figure 4:**
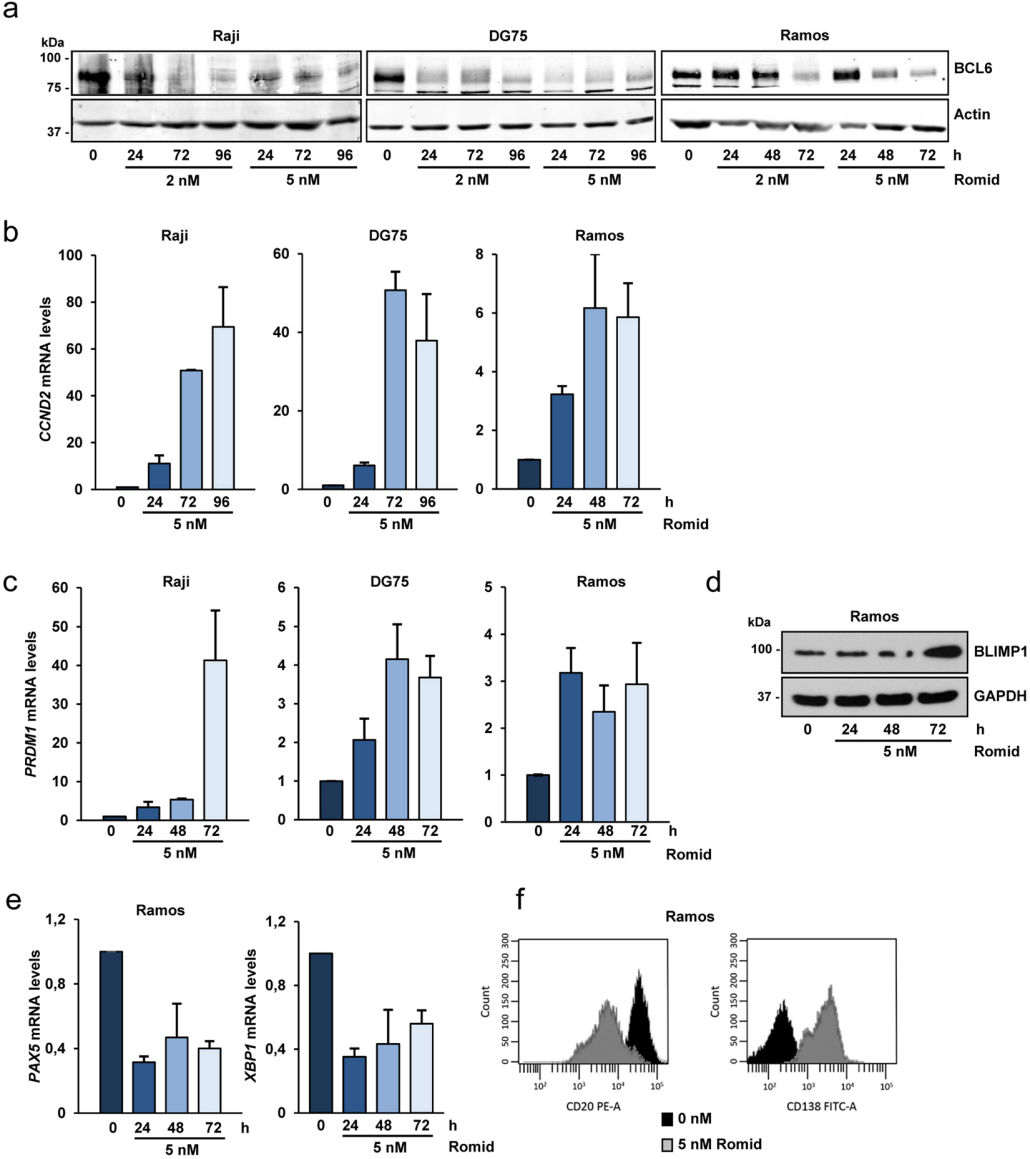
Romidepsin induces BCL6 downregulation and markers of the plasma cell differentiation program. (**a**) Western blot showing BCL6 protein expression in B-cell lymphoma cells treated with 2 or 5 nM romidepsin for the indicated times. Actin was used as loading control. (**b**) RT-PCR showing CCND2 mRNA expression in B-cell lymphoma cells upon romidepsin treatment for the indicated times. mRNA levels were normalized to the ribosomal S14 expression. The data show the means ± s.e.m. of at least two independent experiments. (**c**) RT-PCR showing PRDM1 mRNA expression in B-cell lymphoma cells as in (**b**). (**d**) Western blot showing BLIMP1 protein expression levels in Ramos cells upon romidepsin treatment for the indicated times. (**e**) RT-PCR showing PAX5 and XBP1 mRNA expression levels in Ramos cells as in (**b**). (**f**) Surface markers CD20 and CD138 analyzed by flow cytometry in Ramos cells untreated (black) or treated with 5 nM romidepsin (grey) for 72 h.

BCL6 is a transcriptional repressor and one of the “master regulators” that controls the exit of the B-cells from the germinal centers in order to differentiate toward plasma cells^3^. To determine the effect of romidepsin on *BCL6* expression, we analyze the effect of this histone deacetylase inhibitor on several BL cell lines with different BCL6 expression levels (see Supplementary Table S1). Romidepsin reduced BCL6 mRNA (not shown) and protein levels (Fig. 4a) in a dose and time dependent manner. In DG75, BCL6 downregulation takes place after 24 hours of romidepsin treatment either with 2 nM or 5 nM concentrations. In Raji and Ramos, reduced BCL6 levels were observed upon 48 to 72 hours of treatment. Romidepsin effects on *CCND2* (Cyclin D2), a direct target of BCL6^35^, were evaluated. We found a strong increase in the *CCND2* mRNA levels in Raji and DG75 in response to romidepsin, and a modest upregulation in Ramos cells (Fig. 4b), supporting a functionally important downregulation of BCL6 by romidepsin.

BCL6 downregulation is essential for B-cells to leave the germinal center and to differentiate towards plasma cell. As a consequence of BCL6 downregulation, induction of target genes essential for B-cells to differentiate into plasma cells, such as *PRDM1* (BLIMP1), takes place. We analyzed *PRDM1* mRNA levels in the B-cell lymphoma cell lines upon romidepsin treatment. The gene was upregulated in Raji, DG75 and Ramos cells (Fig. 4c). No significant changes were detected in the non-GC Ly03 cell line (Supplementary Fig. S1). B-cells exit from germinal centers and plasma cell differentiation program not only requires changes in *BCL6* and *PRDM1* expression but also changes in other regulators such as *PAX5* and *XBP1*^3^. We chose the extensively used cell line model Ramos to study the modifications in the expression of these other genes. A clear increase in BLIMP1 protein levels was detected in Ramos cells upon romidepsin treatment (Fig. 4d), accompanied by a decrease in *PAX5* and *XBP1* mRNA expression (Fig. 4e). Finally, surface markers were analyzed by flow cytometry in Ramos cells. The induction of plasma cell differentiation was corroborated by the decrease of CD20 (B-cell marker) and the increase of CD138 (plasma cell marker) (Fig. 4f).

Altogether these results indicate that romidepsin triggers differentiation towards plasma cell differentiation by inhibiting BCL6 expression and this is independent of apoptosis induced by romidepsin.

### Romidepsin induces BCL6 acetylation

**Figure 5:**
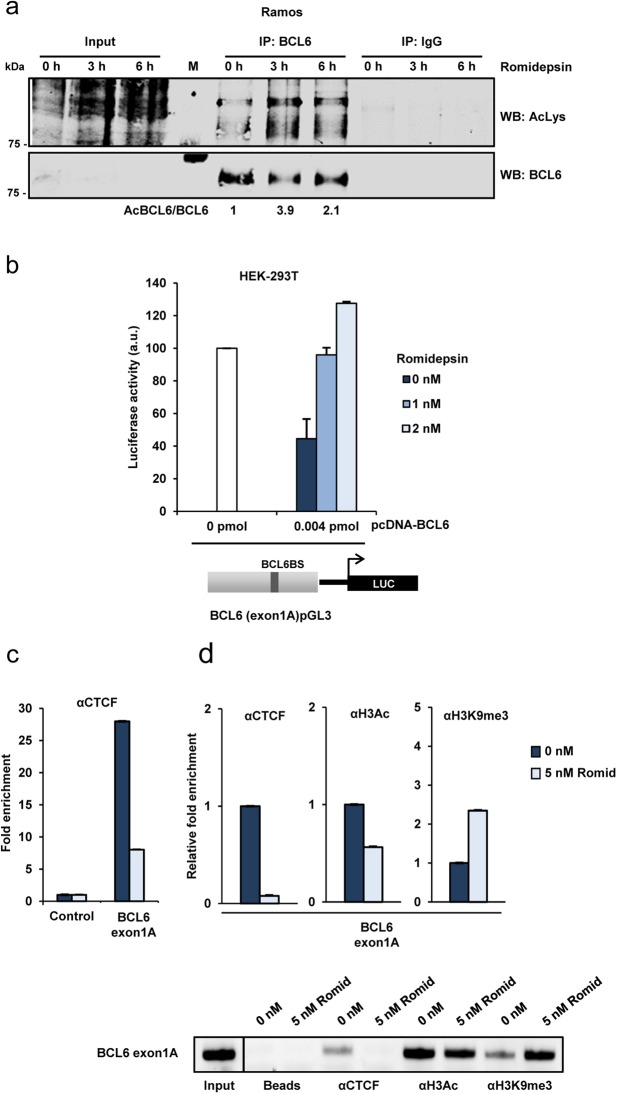
Romidepsin effects on BCL6 acetylation, BCL6 autoregulation and local chromatin structure at the BCL6 exon1A. (**a**) Immunoprecipitation showing BCL6 acetylation. Immunoprecipitation of BCL6 protein in Ramos cells treated for 3 h and 6 h with 10 nM romidepsin. Total lysates were immunoprecipitated with anti-BCL6 antibodies, and the presence of acetylated BCL6 and total amount of BCL6 immunoprecipitated was detected by western blot. Densitometry values are shown at the bottom. Inputs are shown as controls. Immunoprecipitation of IgG was used as negative control. (**b**) Romidepsin effect on the negative autoregulation of BCL6 gene. Luciferase assay showing the effects of romidepsin on BCL6 exon1A regulatory region. HEK-293T cells were co-transfected with either pGL3 vector or with the BCL6 (exon1A)pGL3 reporter vector together with the indicated amount of pCDNA-BCL6 expression vector. pRL-null vector was used to normalize the transfection efficiency. Transfected cells were exposed to the indicated doses of romidepsin for 12 h and luciferase activity was determined 48 h after transfection. The data show the means ± s.e.m. of three measurements in three independent experiments. a.u., arbitrary units. BCL6 gene regulatory region indicating the BCL6 binding site (BCL6BS) is shown. (**c**) Romidepsin effect on CTCF *in vivo* binding to BCL6 exon1A. ChIP analysis with a mix of three anti-CTCF antibodies, showing the binding of this protein to the exon1A of BCL6. Chromatin was prepared from Ramos cells. Relative enrichment was quantified by real-time PCR. The fold enrichment was determined as indicated in Methods section. (**d**) Presence of histone marks at the BCL6 exon1A upon treatment with romidepsin. ChIP analysis with CTCF, H3Ac and H3K9me3 antibodies, showing the presence of these proteins at the exon1A of BCL6. Chromatin was prepared from Ramos cells treated with romidepsin. Relative enrichment was quantified by real-time PCR. The fold enrichment of a particular target sequence was determined as indicated in Methods section. Bottom panel shows typical PCR products after the ChIP assays. The gels were cropped for improved clarity and the full-length gels were included in the Supplementary Information file.

BCL6 regulation is critical for germinal center development. Acetylation inactivates BCL6 either under physiological conditions or induced by the histone deacetylase inhibitor trichostatin A^20^. Although we have shown that long-term romidepsin treatment downregulates BCL6, we wondered whether romidepsin was able to induce BCL6 acetylation at shorter times. Using Ramos germinal center cells as a model, we assayed BCL6 acetylation after romidepsin treatment. Acetylation was assessed by immunoprecipitation with an antibody against BCL6, followed by western blot employing an antibody widely demonstrated to recognize acetylated lysines in several transcription factors or an antibody against BCL6 (Fig. 5a). We found increased BCL6 acetylation (3.9-fold) after 3 hours of romidepsin treatment and this acetylation remains after 6 hours of treatment, although to a lesser extent (2.1-fold) (Fig. 5a top panel). Under all conditions there were approximately equal amounts of BCL6 in each immunoprecipitate (Fig. 5a bottom panel). These results are similar to those previously reported using a different HDACi^20^, demonstrating that romidepsin induces BCL6 acetylation.

### Romidepsin effects on transcription and histone marks at the BCL6 locus

As previously described, BCL6 bound to the *BCL6* exon1A represses its own transcription^7,36,37^. To investigate if the acetylation of BCL6 induced by romidepsin had consequences for transcription from the BCL6 locus, we used luciferase reporter assay in HEK-293T, a cell line that does not express BCL6 (Fig. 5b). We prepared a luciferase reporter construct containing the BCL6 binding site in exon1A, designated as BCL6(exon1A)pGL3. This reporter was cotransfected with a BCL6 expression vector (pCDNA-BCL6). Low luciferase activity was detected in cells transfected with pCDNA-BCL6 in the absence of romidepsin due to the BCL6 negative autoregulation. Luciferase activity progressively increased with romidepsin in a dose-dependent manner (Fig. 5b). Therefore, the negative regulation of BCL6 was counteracted by romidepsin, suggesting that the HDACi is inhibiting BCL6 negative autoregulation by acetylation.

We have previously demonstrated that the chromatin regulator CTCF epigenetically regulates *BCL6* by binding to *BCL6* exon1A^15^. In addition, we have observed that (at timepoints longer than 24 hours) romidepsin reduces BCL6 expression (Fig. 4a). Therefore, we aimed to analyze the CTCF occupancy of the BCL6 exon1A in that context, using ChIP assays in Ramos cells. Results revealed CTCF binding to the BCL6 exon1A in the untreated cells, which strongly diminishes upon treatment with romidepsin (Fig. 5c). Finally, we analyzed the effect of romidepsin on the local chromatin structure at exon1A. Ramos cells were treated with romidepsin and ChIP assays using antibodies against modified histones were performed. A reduction in the binding of the active histone mark H3Ac together with an enrichment of the H3K9me3 repressive histone was observed in the BCL6 exon1A when cells were treated with romidepsin (Fig. 5d). Together these results indicate that romidepsin protects the BCL6 regulatory region against CTCF binding thus favoring the incorporation of repressive histone marks on BCL6 exon1A.

Collectively our results indicate that, at relatively short time-points, romidepsin inhibition of the BCL6 deacetylation presumably repress BCL6 function as shown by others^20^ but increase transcription at the BCL6 locus. At longer time points, romidepsin modifies the local chromatin structure at the BCL6 locus to suppress transcription and consequently BCL6 protein levels, to de-repress transcription of BCL6 target genes.

### Romidepsin and JQ1 synergistic effects on proliferation and apoptosis

**Figure 6:**
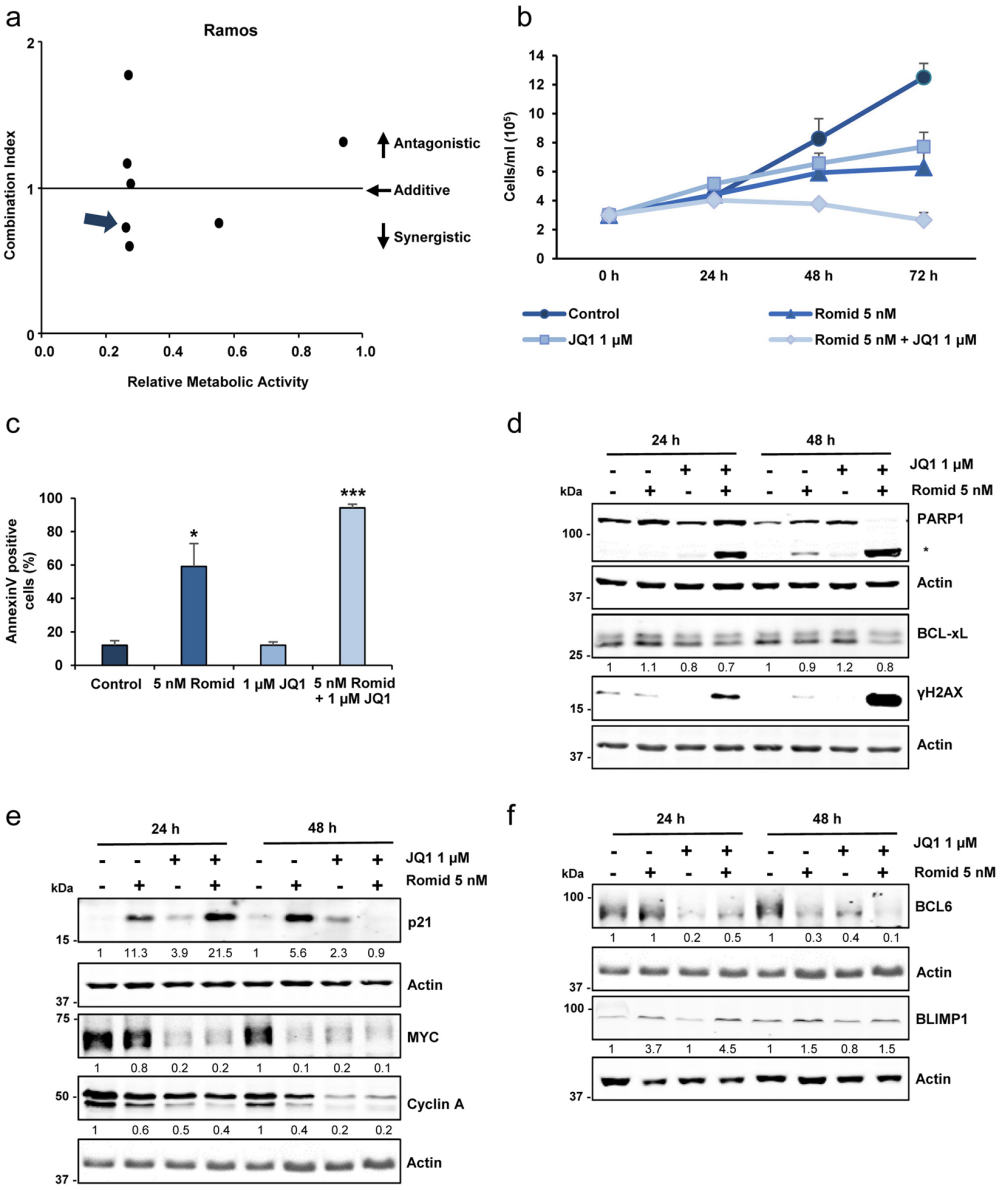
Synergistic effects of romidepsin and JQ1 in Ramos B-cell lymphoma cells. (**a**) Combination index plot showing synergistic effect (arrow) of romidepsin (5 nM) plus JQ1 (1 µM) on the proliferation of Ramos cells. (**b**) Growth curves of Ramos cells untreated (Control) or treated with romidepsin and/or JQ1 for up to 72 h. (**c**) Annexin V staining to assess apoptosis in Ramos cells untreated (Control) or treated with romidepsin and/or JQ1 for 48 h. Results shown are the means ± s.e.m. of three experiments; significance difference (*p < 0.03; ***p < 0.001) from the control untreated cells. (**d**) Western blot showing cleaved PARP1 and BCL-xL and γH2AX protein levels in Ramos cells treated with romidepsin and/or JQ1 for the indicated times. Actin was used as loading control. Densitometry values are shown at the bottom, normalized to the control. (**e**) Western blot showing p21, MYC and cyclin A protein levels in Ramos cells treated as above. (**f**) Western blot showing BCL6 and BLIMP1 protein levels in Ramos cells treated as above. The blots were cropped for improved clarity and the full-length blots were included in the Supplementary Information file.

We next aimed to analyze the effect of combined treatment of the epigenetic drugs romidepsin (HDACi) and JQ1 (BRD4i) in Ramos, a Burkitt lymphoma cell line. Cell viability analysis (WST-1 method) using different concentrations of both compounds were performed to generate combination index (CI) plot (Supplementary Table S2a). For further experiments, we choose doses close to the IC_50_ values (5 nM romidepsin and 1 µM JQ1) where synergism (CI < 1) was evident (Fig. 6a). Growth curve analysis confirmed the synergistic effect on cell proliferation (Fig. 6b). Annexin V staining (Fig. 6c) and cleaved PARP1 (Fig. 6d) were strongly increased after 48 h with the combination treatment, together with decreased levels of the antiapoptotic protein BCL-xL, indicating significant synergistic effect of romidepsin and JQ1 on apoptotic cell death. Interestingly, the combination treatment dramatically induced the expression of γH2AX (Fig. 6d), a well-known marker of DNA damage response.

We also analyzed the effects of romidepsin and JQ1 on the cell cycle. Both compounds alone provoked cell cycle arrest and increased the expression of p21 (Fig. 6e). A further increase in p21 levels was detected after 24 h with the combination treatment. At longer times, this synergistic effect was not evident presumably due to the high proportion of apoptotic cells (Fig. 6c). The expected decrease in the cyclin A (as a marker of proliferation) and MYC levels was also observed (Fig. 6e). Finally, we found downregulation of BCL6 expression that was more pronounced upon 48 h of combination treatment while BLIMP1 levels were slightly increased with both romidepsin and JQ1 (Fig. 6f).

**Figure 7:**
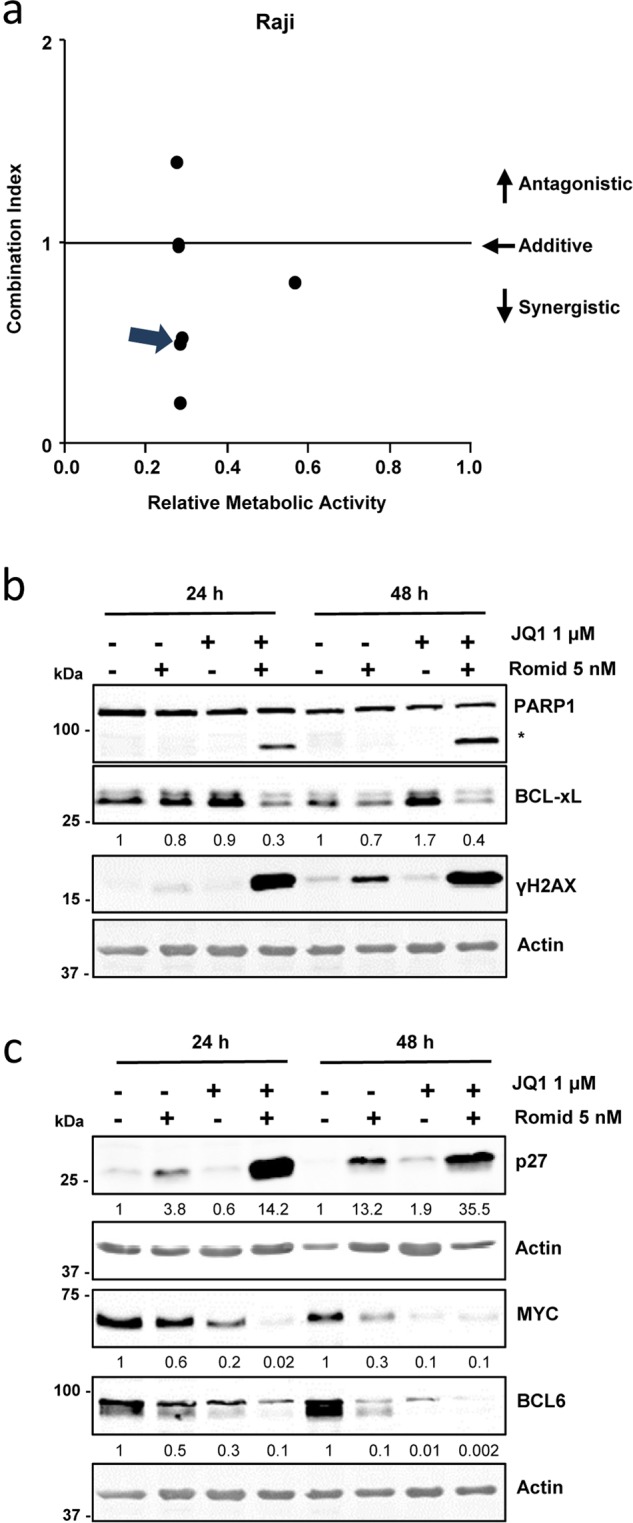
Synergistic effects of romidepsin and JQ1 in Raji B-cell lymphoma cells. (**a**) Combination index plot showing synergistic effect (arrow) of romidepsin (5 nM) plus JQ1 (1 µM) on the proliferation of Raji cells. (**b**) Western blot showing cleaved PARP1 and BCL-xL and γH2AX protein levels in Raji cells treated with romidepsin and/or JQ1 for the indicated times. Actin was used as loading control. Densitometry values are shown at the bottom, normalized to the control. (**c**) Western blot showing p27, MYC and BCL6 protein levels in Raji cells treated as above. The blots were cropped for improved clarity and the full-length blots were included in the Supplementary Information file.

Similar results were obtained in the less sensitive Raji cells from BL as shown by the CI plot (Fig. 7a, Supplementary Table S2b), and to a lesser extent in Toledo and DG75 cells (Supplementary Figs S2 and S3). Clear synergistic effects of romidepsin and JQ1 in terms of apoptosis (PARP1 cleavage and γH2AX induction) (Fig. 7b), cell cycle arrest (p27 induction, MYC downregulation) and plasmatic differentiation (BCL6 downregulation) (Fig. 7c) were observed, indicating that the drugs combination could be effective in aggressive B cells lymphoma.

## Discussion

Novel strategies for treatment of aggressive lymphomas are needed. DLBCL, one of the most frequent lymphomas in western countries will be refractory to anthracycline conventional treatments in a substantial proportion of cases. BCL6 is a key regulator of normal germinal center B-cells and is expressed in Burkitt lymphoma and some DLBCL. Transcription factors are attractive targets for therapy because they control programs of gene expression^38^. It is likely that the gene expression program controlled by BCL6 has important roles in driving cell cycle progression and proliferation in normal and malignant B-cells^35,39^ in turn suggesting that BCL6 is an attractive target for these types of lymphomas^10,12,13^. Inhibition of BCL6 has indeed shown very promising results in preclinical studies^40^. Moreover, targeting BCL6 might not only be effective on germinal center derived lymphomas but also those ABC-DLBCL cases that express it^41^. However, generation of potent, drug-like inhibitors against BCL6 remains challenging.

We and others, have shown that different epigenetic mechanisms are involved in the modulation of BCL6 expression in the germinal center reaction^14,15^. Epigenetic modulators are an attractive and effective strategy in a number of malignancies^17^. Among them, more than ten histone deacetylase inhibitors have shown promising results in studies performed in different hematological malignances, including lymphomas^18,21,34^. HDACi can produce a variety of effects including induction of apoptosis, cell cycle arrest, induction of differentiation and regulation of immune responses, and these effects are exerted in a cell type specific manner^21,22,34,42^. Some of these effects are generated by the inhibition of activity of HDACs while others might be the consequence of modification of other cellular proteins by acetylation. In this study a number of BL derived cell lines were used to evaluate the effect of romidepsin, a potent HDACi that has been approved for treatment of different types of T cell lymphomas but its role on B cell lymphomas is yet not well known. BL shows high level BCL6 expression and BL cell lines have been used to elucidate many aspects of BCL6 action^35^. We also analyzed the effect of romidepsin in two GC-DLBCL and ABC-DLBCL cell lines, for comparison.

Romidepsin induced apoptosis in metabolically sensitive lymphoma B-cell lines. Several reports have shown a role for the mitochondrial apoptotic pathway in HDACi mediated apoptosis^21,42^. In some cases BCL2 or BCL-xL, which block the intrinsic apoptotic pathway, are able to overcome the effects of a number of HDACi. Therefore, a differential expression of the pro-apoptotic and the anti-apoptotic BCL2 family members might explain the varying response to romidepsin. In healthy B-cells, BCL2 expression is reduced when B-cells enter the germinal center reaction^43^. Although the basal expression of BCL2 was variable among the cells used in this study, no relationship between BCL2 expression and sensitiveness to romidepsin was observed. This is not surprisingly since romidepsin has been shown to induce apoptosis even in models overexpressing BCL2, but not in BCL-xL models^33^. Although down-regulation of BCL-xL does not imply an immediate death^43^, the reduction in the anti-apoptotic BCL-xL in Ramos and Toledo cells compared to the unchanged levels observed in the DG75 insensitive cell line, imply that BCL-xL might be a good marker for monitoring romidepsin effect in germinal center cells. This is in agreement with previous studies showing how expression of BCL-xL might confer a drug resistance phenotype^44^. Interestingly, BIM was upregulated in both DLBCL (Toledo and Ly03) cells in the presence of romidepsin, which might explain the apoptosis observed in those cell lines, given that BIM plays a critical role in HDACi induced apoptosis^45,46^. In DG75 and Ramos cells, BIM levels are maintained while MCL1 protein transiently increases upon romidepsin treatment. Since MCL1 is essential for the survival of plasma cells^47^, this transient expression of MCL1 detected in response to romidepsin might be providing a short-term window to allow cells to differentiate into plasma cells and/or die by apoptosis.

It is known that GC B-cells stop proliferating in order to be able to differentiate into plasma cells. Accordingly, cell cycle arrest in G_0_/G_1_ phase was observed, together with the increase in p21 and p27, in most of the lymphoma cells analyzed upon exposure to romidepsin. This is in line with previous reports showing, that accumulation of cycling dependent kinase inhibitors is a general effect shared by most of all the histone deacetylase inhibitors, when used on various malignancies^19,34^.

**Figure 8:**
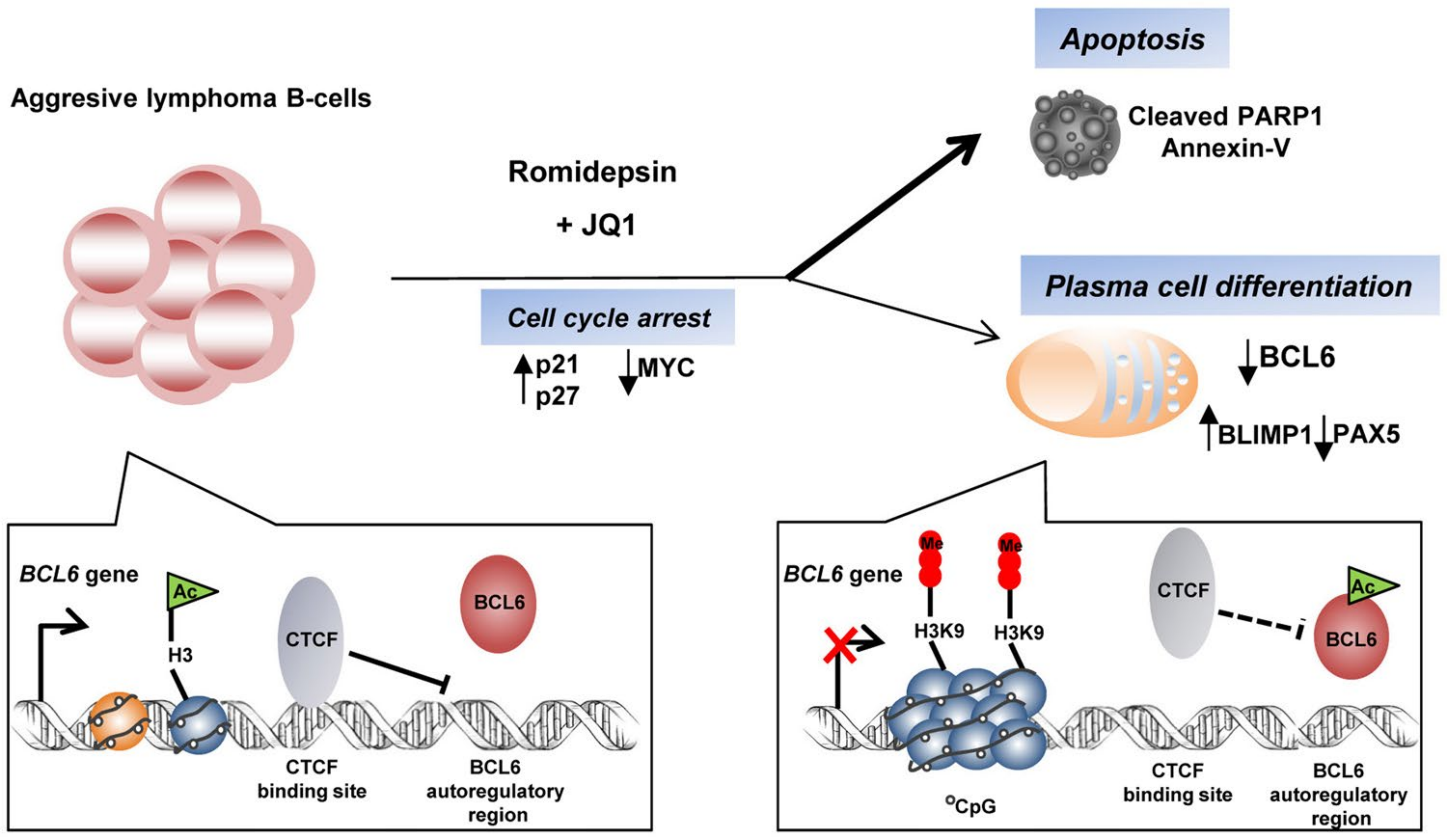
Proposed model for epigenetic drugs effect on B-cell lymphoma cells and BCL6 regulation. Romidepsin induces cell cycle arrest accompanied with increased levels of p27 and p21 in most lymphoma cells. The sensitive lymphoma B-cells undergo apoptosis as shown by cleavage of PARP1 and positive Annexin V staining. Downregulation of BCL6 and PAX5 together with increase on PRDM1 (BLIMP1) is also observed, and plasma cell differentiation program is initiated. Synergistic effect of romidepsin and JQ1 mainly in apoptosis induction (thicker line) is demonstrated. In B-cell lymphoma cells, CTCF is bound to BCL6 exon1A impairing BCL6 binding to its negative autoregulation site and is associated with marks representative of an open chromatin state (H3Ac). In the presence of romidepsin BCL6 is acetylated, CTCF is not longer bound to BCL6 exon1A and repressive histone marks (H3K9me3) are incorporated. The consequent downregulation of BCL6 correlates with plasmatic differentiation as shown above.

In this study we have shown that romidepsin (i) induces BCL6 acetylation, which inhibits BCL6 negative autoregulation, and (ii) protects the BCL6 regulatory region against CTCF binding thus favouring the incorporation of repressive histone marks. In agreement, treatment of DLBCLs with other HDACi has been shown to induce hyperacetylation of BCL6, reactivation of repressed target genes and induction of apoptosis^20,48^. In our study, the romidepsin-mediated downregulation of BCL6 was also associated with plasma cell differentiation, as shown by induction of PRDM1 (BLIMP1) levels and the expression of plasma cell surface marker (CD138). In treated Ramos cells, *PAX5* mRNA levels were downregulated, PAX5 being necessary for B-cells to maintain their germinal center identity^49,50^. However, when we analyzed the expression of XBP1, a gene that acts downstream of PRDM1-BLIMP1, we found no upregulation, suggesting that romidepsin might be triggering a partial plasma cell differentiation program but is not sufficient for terminal plasma cell differentiation. Therefore, as expected the mature plasma cell phenotype was not completely reached because cell lines have limited differentiation capacity. The effects of romidepsin on lymphoma B cells are summarized in Fig. 8.

Combination of HDACi with drugs targeting different cellular pathways are being used with promising results in a number of tumors including lymphomas^18,19^. Recently, combination treatments of HDAC and BET inhibitors have been described to have synergistic effects in different cancers^51–61^. In order to further improve the response of aggressive lymphoma cells, we explored the effects of romidepsin together with the BRD4 inhibitor JQ1. BRD4i inhibits *MYC* expression, frequently deregulated in hematopoietic malignancies^28,62^. Strong synergistic effect of romidepsin and JQ1 activating DNA-damage response and apoptotic cell death was found in Ramos cells (from BL) as assessed by γH2AX induction, annexin V binding and PARP1 cleavage. Notably, synergy in apoptosis and plasma cell differentiation was also found in the less sensitive Raji and DG75 (from BL) and Toledo (from GC-DLBCL) cells. In agreement with our results, two recent reports demonstrated synergistic effects of romidepsin and JQ1 in solid tumors^55,56^. This novel combination therapy can be useful to treat poor prognosis or non-responders low survival lymphoma patients.

In conclusion, our findings provide new insights into the molecular mechanisms of romidepsin effect on aggressive lymphomas: cell cycle arrest and induction of plasma differentiation is induced upon exposure to the drug and this effect appear to be related to the inhibition of BCL6 expression and function. Romidepsin also induces a variable apoptotic effect that is significantly increased upon treatment with the BET inhibitor JQ1, which reduces MYC function. Overall our data show a potential role for romidepsin and JQ1 combination for the treatment of aggressive and BCL6 expressing lymphomas.

## Methods

### Cell lines culture and drugs treatment

Raji, DG75, Ramos, Toledo and Ly03 B-cell lymphoma cell lines (origin and features in Supplementary Table S1) were grown in RPMI-1640 supplemented with 10% or 20% fetal calf serum (Lonza) under standard conditions^15^ and confirmed to be mycoplasma free. *BCL6*, *MYC* and *BCL2* loci status in the studied cell lines were determined by FISH analysis (Supplementary Table S1).

Romidepsin was kindly provided by Celgene (Summit, NJ, USA). (+)-JQ1 was purchase from Cayman Chemical (Ann Arbor, MI, USA). Drugs were diluted in DMSO and stored at −20 °C. To study the effects of the drugs, exponentially growing cells were treated with the different drug concentrations for several time points, depending on the experiment and cell line.

### Cell proliferation and viability assays

Cells were treated with different doses of romidepsin and/or JQ1 for up to 96 h. Cell proliferation and cell viability was measured using the Guava ViaCount reagent (Merck Millipore, Darmstadt, Germany) or the NucleoCounter system (Chemometec, Allerod, Denmark). Metabolic activity of cells was measured using the WST-1 method (Roche, Basilea, Switzerland) which allows the quantification of the number of viable cells by the cleavage of tetrazolium salt WST-1 to formazan dye. Romidepsin and JQ1 combination effects were determined using the combination index (CI) values, analyzed by the Chou-Talalay method using CompuSyn Software^63^.

### Cell cycle, apoptosis and differentiation analysis

Cell cycle analysis were performed using propidium iodide staining as previously described^64^. Cells were fixed with cold ethanol and resuspended in PBS-citrate Na-BSA containing RNase and propidium iodide. The stained cells were analyzed by flow cytometry (FACSDiva cytometer). Annexin V-PE Apoptosis detection Kit (Immunostep, Salamanca, Spain) was used for the detection of early apoptotic cells. Cells were treated with romidepsin and JQ1 for 24 and 48 h and Annexin V-binding was analyzed by flow cytometry (FACSDiva^TM^ software, BD biosciences, NJ, USA). The cleavage of poly(ADP-ribose)polymerase-1 (PARP1), indicative of apoptosis, was analyze by immunoblot.

Cell surface markers were analyzed in the Hematology Department of Hospital Marqués de Valdecilla to assess cells differentiation. After 72 h of culture in the presence of 5 nM romidepsin, cell surface markers were analyzed by flow cytometry using the BD FACSDiva flow-cytometer following standard procedures^65^. The conjugated antibodies used were: anti-CD20 PE (BD 345793) from BD Biosciences and anti-CD138 FITC (Rafer; Zaragoza, Spain, IQP 153 F).

### RNA analysis by Reverse transcription (RT) and polymerase chain reaction (PCR)

Total RNA was isolated by using the Trizol reagent (Invitrogen, Carlsbad, CA, USA). For reverse transcription, first-strand cDNA was synthesized from 1 µg of total RNA using the iScript cDNA Synthesis Kit (Bio-Rad, Hercules, CA, USA). Quantitative PCR was performed with a IQTM SyBR Green Supermix kit (Bio-Rad). mRNA expression was normalized to ribosomal protein S14 mRNA levels (primers shown in Supplementary Table S3). Results were analyzed by comparative ΔΔCt method and expressed as mean ± SEM of duplicate PCRs from at least two independent experiments.

### Immunoblot and immunoprecipitation assays

Cells were lysed in lysis buffer (1% NP-40, 100 mM NaCl, 20 mM Tris-HCl pH 7.4, 10 mM NaF, 1 mM orthovanadate and protease and phosphatase inhibitors cocktail). Samples were sonicated and subjected to SDS-PAGE and immunoblot as described previously^66^. The antibodies used were: anti-actin (I-19, sc1616), anti-BCL6 (N-3, sc858), anti-PARP1 (H-250, sc7150), anti-CycA (H-432, sc751) from Santa Cruz Biotech. (Santa Cruz, CA, USA); anti-BCL-xL (ab7974) from Abcam (Cambrigde, UK); anti-BCL2 (2876), anti-BIM (C34C5), anti-MCL1 (4572), anti-BLIMP1 (C14A4), anti-GAPDH (14C10), anti-cMYC (9402S), anti-p27 (3686), anti-p21 (2947) from Cell Signalling Tech. (Danvers, MA, USA); Anti-phospho-Histone H2A.X (Ser139) (05–636) from Millipore (Darmstadt, Germany). Blots were developed with secondary antibodies conjugated to IRDye680 or IRDye800 (Li-Cor Biosciences, LiCor, Lincoln, NE, USA) and immunocomplexes were detected with an Odyssey infrared-imaging system (Li-Cor Biosciences). Some blots were revealed with the ECL system (Bio-Rad).

Immunoprecipitations were performed essentially as described^67^. For detection of the endogenous acetylated BCL6, Ramos cells were lysed in lysis buffer (50 mM Tris-HCl, 250 mM NaCl, 1% Nonidet P-40, 0.5% deoxycholic acid, 0.1% SDS) containing 1 μM romidepsin and protease inhibitors cocktail. Protein extracts were immunoprecipitated with 3 μg of a mouse monoclonal antibody against BCL6 (PG-B6P) or unspecific immunoglobulins used as a control (IgGs sc-2025), from Santa Cruz Biotech. Dynabeads-protein G-bound magnetic beads (Invitrogen) were used to capture protein-antibody immunocomplexes. Immunocomplexes were resolved by SDS-PAGE and analyzed by western blot with antibody against acetyl lysine (9441, Cell Signaling). The same filter was then incubated with an anti-BCL6 (N3, sc858) rabbit polyclonal antibody.

### Luciferase reporter assays

HEK-293T cells were transiently transfected using polyethilenimine (PEI) transfection reagent (Polysciences Inc., Warrington, PA, USA) as previously described^15^ and reporter experiments were performed essentially as described^20^. 0.3 µg of the pGL3-basic vector (Promega) or 0.35 µg of the BCL6(exon1)-pGL3 reporter vector^68^ and 0.1 µg of the pRL-null vector (Promega) were cotransfected with pCDNA-BCL6 expression vector^15^. Cells were treated for 12 hours with different concentrations of romidepsin. Luciferase activities were measured 48 hours after transfection using the Dual-Glo Luciferase Reporter System (Promega, Madison, WI, USA) in a Luminometer TD 20/20 Turner Designs. For each determination, luciferase activity was calculated as the Firefly activity normalized by the Renilla activity. Luciferase activity in arbitrary units (a.u.) was shown as the increase in activation relative to the activity of the pGL3 vector alone and the maximum value for each condition was set to 100.

### Chromatin immunoprecipitation (ChIP)

For ChIP experiments chromatin was prepared from Ramos cells treated with 5 mM romidepsin for 48 h. ChIP assays were performed using the Pierce Magnetic ChIP Kit (Thermo Fisher Scientific, Waltham, MA, USA) following the manufacturer’s protocol. Cells were fixed in formaldehyde, lysed, treated with Micrococcal nuclease and sonicated using a Bioruptor UCD-200TM (Diagenode, Liège, Belgium). ChIP was performed using ChIP-Grade Protein A/G Magnetic Beads coupled to different antibodies: anti-CTCF-Ab-1 (07-729) from Millipore, anti-CTCF (ab10571) from Abcam; anti-CTCF (612149) from BD and anti-H3acetylated (06-599) from Millipore; anti-H3K9me3 (ab8898) from Abcam. Real-time PCR of immunoprecipitated DNA was performed in duplicate with equal amounts of specific antibody immunoprecipitated sample, control (beads) and input. Primers used for ChIP assays corresponding to the BCL6 exon 1A (+257) were: 5′-GCACTCCCCCTCTTATGTCA-3′ and 5′-GATTTGGAGGTTCCGGTTC-3′^15^. The comparative cycle threshold approach was used for the data analysis^15^. The signals were normalized to the inputs and the fold enrichment was calculated relative to the control sample (no-antibody). The values are the mean ± S.E.M. of two to six independent experiments. Normalization of histone marks was based on the ChIP-IT qPCR analysis kit (Active motif North America, Carlsbad, CA, USA).

### Statistical analysis

Results were presented as the mean of two to four determinations with error bars representing the standard error of the mean. The significance of differences was determined by the unpaired Student’s *t* test; a *p* < 0.05 was considered to be statistically significant.

## Supplementary information

Supplementary information

## References

[1] Swerdlow SH (2016). The 2016 revision of the World Health Organization classification of lymphoid neoplasms. Blood.

[2] Coiffier B (2010). Long-term outcome of patients in the LNH-98.5 trial, the first randomized study comparing rituximab-CHOP to standard CHOP chemotherapy in DLBCL patients: a study by the Groupe d’Etudes des Lymphomes de l’Adulte. Blood.

[3] Basso K, Dalla-Favera R (2015). Germinal centres and B cell lymphomagenesis. Nat Rev Immunol.

[4] Dent AL, Shaffer AL, Yu X, Allman D, Staudt LM (1997). Control of inflammation, cytokine expression, and germinal center formation by BCL-6. Science.

[5] Ye BH (1997). The BCL-6 proto-oncogene controls germinal-centre formation and Th2-type inflammation. Nat Genet.

[6] Cattoretti G (2005). Deregulated BCL6 expression recapitulates the pathogenesis of human diffuse large B cell lymphomas in mice. Cancer Cell.

[7] Pasqualucci L (2003). Mutations of the BCL6 proto-oncogene disrupt its negative autoregulation in diffuse large B-cell lymphoma. Blood.

[8] Hatzi K, Melnick A (2014). Breaking bad in the germinal center: how deregulation of BCL6 contributes to lymphomagenesis. Trends Mol Med.

[9] Ye BH, Rao PH, Chaganti RS, Dalla-Favera R (1993). Cloning of bcl-6, the locus involved in chromosome translocations affecting band 3q27 in B-cell lymphoma. Cancer research.

[10] Cardenas MG (2017). The Expanding Role of the BCL6 Oncoprotein as a Cancer Therapeutic Target. Clin Cancer Res.

[11] Wagner SD, Ahearne M, Ko Ferrigno P (2011). The role of BCL6 in lymphomas and routes to therapy. Br J Haematol.

[12] Leeman-Neill RJ, Bhagat G (2018). BCL6 as a therapeutic target for lymphoma. Expert opinion on therapeutic targets.

[13] Pasqualucci L (2019). Molecular pathogenesis of germinal center-derived B cell lymphomas. Immunological reviews.

[14] Lai AY (2010). DNA methylation prevents CTCF-mediated silencing of the oncogene BCL6 in B cell lymphomas. The Journal of experimental medicine.

[15] Batlle-Lopez A (2015). Novel CTCF binding at a site in exon1A of BCL6 is associated with active histone marks and a transcriptionally active locus. Oncogene.

[16] Batlle-Lopez A, Cortiguera MG, Delgado MD (2015). The epigenetic regulator CTCF modulates BCL6 in lymphoma. Oncoscience.

[17] Dawson MA, Kouzarides T (2012). Cancer epigenetics: from mechanism to therapy. Cell.

[18] Ahuja N, Sharma AR, Baylin SB (2016). Epigenetic Therapeutics: A New Weapon in the War Against Cancer. Annual review of medicine.

[19] New M, Olzscha H, La Thangue NB (2012). HDAC inhibitor-based therapies: can we interpret the code?. Molecular oncology.

[20] Bereshchenko OR, Gu W, Dalla-Favera R (2002). Acetylation inactivates the transcriptional repressor BCL6. Nat Genet.

[21] West AC, Johnstone RW (2014). New and emerging HDAC inhibitors for cancer treatment. The Journal of clinical investigation.

[22] Bates SE, Robey RW, Piekarz RL (2015). CCR 20th Anniversary Commentary: Expanding the Epigenetic Therapeutic Portfolio. Clin Cancer Res.

[23] Kalac M (2011). HDAC inhibitors and decitabine are highly synergistic and associated with unique gene-expression and epigenetic profiles in models of DLBCL. Blood.

[24] Aukema SM (2014). Biological characterization of adult MYC-translocation-positive mature B-cell lymphomas other than molecular Burkitt lymphoma. Haematologica.

[25] Gupta M (2012). Expression of Myc, but not pSTAT3, is an adverse prognostic factor for diffuse large B-cell lymphoma treated with epratuzumab/R-CHOP. Blood.

[26] Delmore JE (2011). BET bromodomain inhibition as a therapeutic strategy to target c-Myc. Cell.

[27] Filippakopoulos P (2010). Selective inhibition of BET bromodomains. Nature.

[28] Cortiguera MG, Batlle-López A, Albajar M, Delgado MD, León J (2015). MYC as therapeutic target in leukemia and lymphoma. Blood and Lymphatic. Cancer: Targets and Therapy.

[29] Andrieu G, Belkina AC, Denis GV (2016). Clinical trials for BET inhibitors run ahead of the science. Drug discovery today. Technologies.

[30] Peart MJ (2003). Novel mechanisms of apoptosis induced by histone deacetylase inhibitors. Cancer research.

[31] Finke J (1992). Expression of bcl-2 in Burkitt’s lymphoma cell lines: induction by latent Epstein-Barr virus genes. Blood.

[32] Ierano C (2013). Loss of the proteins Bak and Bax prevents apoptosis mediated by histone deacetylase inhibitors. Cell Cycle.

[33] Newbold A (2008). Characterisation of the novel apoptotic and therapeutic activities of the histone deacetylase inhibitor romidepsin. Mol Cancer Ther.

[34] Zain J, O’Connor OA (2010). Targeting histone deacetyalses in the treatment of B- and T-cell malignancies. Investigational new drugs.

[35] Shaffer AL (2000). BCL-6 represses genes that function in lymphocyte differentiation, inflammation, and cell cycle control. Immunity.

[36] Kikuchi M (2000). Identification of negative regulatory regions within the first exon and intron of the BCL6 gene. Oncogene.

[37] Wang X, Li Z, Naganuma A, Ye BH (2002). Negative autoregulation of BCL-6 is bypassed by genetic alterations in diffuse large B cell lymphomas. Proceedings of the National Academy of Sciences of the United States of America.

[38] Melnick AM, Adelson K, Licht JD (2005). The theoretical basis of transcriptional therapy of cancer: can it be put into practice?. J Clin Oncol.

[39] Bunting KL (2016). Multi-tiered Reorganization of the Genome during B Cell Affinity Maturation Anchored by a Germinal Center-Specific Locus Control Region. Immunity.

[40] Cerchietti LC (2010). A small-molecule inhibitor of BCL6 kills DLBCL cells *in vitro* and *in vivo*. Cancer Cell.

[41] Cardenas MG (2016). Rationally designed BCL6 inhibitors target activated B cell diffuse large B cell lymphoma. The Journal of clinical investigation.

[42] Bolden JE, Peart MJ, Johnstone RW (2006). Anticancer activities of histone deacetylase inhibitors. Nature reviews.

[43] Peperzak V, Slinger E, Ter Burg J, Eldering E (2017). Functional disparities among BCL-2 members in tonsillar and leukemic B-cell subsets assessed by BH3-mimetic profiling. Cell Death Differ.

[44] Minn AJ, Rudin CM, Boise LH, Thompson CB (1995). Expression of bcl-xL can confer a multidrug resistance phenotype. Blood.

[45] Zhao Y (2005). Inhibitors of histone deacetylases target the Rb-E2F1 pathway for apoptosis induction through activation of proapoptotic protein Bim. Proceedings of the National Academy of Sciences of the United States of America.

[46] Ding H (2017). Histone deacetylase inhibitors interrupt HSP90*RASGRP1 and HSP90*CRAF interactions to upregulate BIM and circumvent drug resistance in lymphoma cells. Leukemia.

[47] Peperzak V (2013). Mcl-1 is essential for the survival of plasma cells. Nat Immunol.

[48] Amengual JE (2013). Sirtuin and pan-class I/II deacetylase (DAC) inhibition is synergistic in preclinical models and clinical studies of lymphoma. Blood.

[49] Revilla IDR (2012). The B-cell identity factor Pax5 regulates distinct transcriptional programmes in early and late B lymphopoiesis. EMBO J.

[50] Nera KP (2006). Loss of Pax5 promotes plasma cell differentiation. Immunity.

[51] Bhadury J (2014). BET and HDAC inhibitors induce similar genes and biological effects and synergize to kill in Myc-induced murine lymphoma. Proceedings of the National Academy of Sciences of the United States of America.

[52] Mazur PK (2015). Combined inhibition of BET family proteins and histone deacetylases as a potential epigenetics-based therapy for pancreatic ductal adenocarcinoma. Nature medicine.

[53] Mishra VK (2017). Histone deacetylase class-I inhibition promotes epithelial gene expression in pancreatic cancer cells in a BRD4- and MYC-dependent manner. Nucleic acids research.

[54] Enssle JC (2018). Co-targeting of BET proteins and HDACs as a novel approach to trigger apoptosis in rhabdomyosarcoma cells. Cancer Lett.

[55] Jostes S (2017). The bromodomain inhibitor JQ1 triggers growth arrest and apoptosis in testicular germ cell tumours *in vitro* and *in vivo*. J Cell Mol Med.

[56] Holscher AS, Schulz WA, Pinkerneil M, Niegisch G, Hoffmann MJ (2018). Combined inhibition of BET proteins and class I HDACs synergistically induces apoptosis in urothelial carcinoma cell lines. Clinical epigenetics.

[57] Borbely G, Haldosen LA, Dahlman-Wright K, Zhao C (2015). Induction of USP17 by combining BET and HDAC inhibitors in breast cancer cells. Oncotarget.

[58] Heinemann A (2015). Combining BET and HDAC inhibitors synergistically induces apoptosis of melanoma and suppresses AKT and YAP signaling. Oncotarget.

[59] Loosveld M (2014). Therapeutic targeting of c-Myc in T-cell acute lymphoblastic leukemia, T-ALL. Oncotarget.

[60] Zhao L, Okhovat JP, Hong EK, Kim YH, Wood GS (2019). Preclinical Studies Support Combined Inhibition of BET Family Proteins and Histone Deacetylases as Epigenetic Therapy for Cutaneous T-Cell Lymphoma. Neoplasia.

[61] Liu S (2019). BRD4 inhibitor and histone deacetylase inhibitor synergistically inhibit the proliferation of gallbladder cancer *in vitro* and *in vivo*. Cancer Sci.

[62] Delgado, M. D., Albajar, M., Gomez-Casares, M. T., Batlle, A. & Leon, J. MYC oncogene in myeloid neoplasias. Clinical & translational oncology: official publication of the Federation of Spanish Oncology Societies and of the National Cancer Institute of Mexico 15, 87–94 (2013). 10.1007/s12094-012-0926-8 22911553

[63] Chou TC, Talalay P (1984). Quantitative analysis of dose-effect relationships: the combined effects of multiple drugs or enzyme inhibitors. Advances in enzyme regulation.

[64] Albajar M (2011). MYC in chronic myeloid leukemia: induction of aberrant DNA synthesis and association with poor response to imatinib. Molecular cancer research: MCR.

[65] Colorado M (2010). Simultaneous cytomorphologic and multiparametric flow cytometric analysis on lymph node samples is faster than and as valid as histopathologic study to diagnose most non-Hodgkin lymphomas. Am J Clin Pathol.

[66] Torrano V (2006). Targeting of CTCF to the nucleolus inhibits nucleolar transcription through a poly(ADP-ribosyl)ation-dependent mechanism. Journal of cell science.

[67] Caraballo JM (2014). High p27 protein levels in chronic lymphocytic leukemia are associated to low Myc and Skp2 expression, confer resistance to apoptosis and antagonize Myc effects on cell cycle. Oncotarget.

[68] Papadopoulou V, Postigo A, Sanchez-Tillo E, Porter AC, Wagner SD (2010). ZEB1 and CtBP form a repressive complex at a distal promoter element of the BCL6 locus. The Biochemical journal.

