# Supplementary material for: Suppression of BCL6 function by HDAC inhibitor mediated acetylation and chromatin modification enhances BET inhibitor effects in B-cell lymphoma cells
Source: Sci Rep. 2019 Nov 11;9:16495. doi: 10.1038/s41598-019-52714-4 (PMC6848194; doi:10.1038/s41598-019-52714-4)
Supplement: Supplementary file 1 — Supplementary information [file 41598_2019_52714_MOESM1_ESM.pdf]

**Suppression of BCL6 function by HDAC inhibitor mediated acetylation and chromatin modification enhances BET inhibitor effects in B-cell lymphoma cells**

María G. Cortiguera[1,2,4], Lorena García-Gaipo[1,4], Simon D. Wagner[3], Javier León[1], Ana Batlle-López[2*] and M. Dolores Delgado[1*].

[1]Instituto de Biomedicina y Biotecnología de Cantabria (IBBTEC) CSIC-Universidad de Cantabria, and Dpt. of. Biología Molecular, Universidad de Cantabria, Santander, Spain.

[2]Servicio de Hematologia, Hospital Marqués de Valdecilla-IDIVAL, Santander, Spain.

[3]Leicester Cancer Research Centre and Ernest and Helen Scott Haematological Research Unit, University of Leicester, Leicester LE1 7HB, UK

[4]Both authors contributed equally to this work

*Corresponding authors

Prof. M. Dolores Delgado, maria.delgado@unican.es

Dr. Ana Batlle-López, mana.batlle@scsalud.es

**Supplementary information**

**Supplementary Table S1.** B-cell lymphoma cell lines used in this study.

**Supplementary Table S2.** Cell proliferation analysis (WST-1 method) in Ramos and Raji cells using different concentrations of Romidepsin and JQ1 to generate Combination Index (CI) plot.

**Supplementary Table S3.** Primers used for RT-PCR analysis.

**Supplementary Figure S1.** Romidepsin effects on BCL6 and PRDM1 expression in Ly03 cells.

**Supplementary Figure S2.** Synergistic effects of romidepsin and JQ1 in Toledo cells.

**Supplementary Figure S3.** Synergistic effects of romidepsin and JQ1 in DG75 cells.

**Supplementary Figure S4.** Full-length blots corresponding to Figure 1c.

**Supplementary Figure S5.** Full-length blots corresponding to Figure 2a.

**Supplementary Figure S6.** Full-length blots corresponding to Figure 2b.

**Supplementary Figure S7.** Full-length blots corresponding to Figure 2c.

**Supplementary Figure S8.** Full-length blots corresponding to Figure 2d.

**Supplementary Figure S9.** Full-length blots corresponding to Figure 3.

**Supplementary Figure S10.** Full-length blots corresponding to Figure 4.

**Supplementary Figure S11.** Full-length blots corresponding to Figure 5.

**Supplementary Figure S12.** Full-length blots corresponding to Figure 6d.

**Supplementary Figure S13.** Full-length blots corresponding to Figure 6e, f.

**Supplementary Figure S14.** Full-length blots corresponding to Figure 7.

**Supplementary Figure S15.** Full-length blots corresponding to Figure S1a.

**Supplementary Figure S16.** Full-length blots corresponding to Figure S2b.

**Supplementary Figure S17.** Full-length blots corresponding to Figure S2c.

**Supplementary Figure S18.** Full-length blots corresponding to Figure S3.

**Supplementary Table S1. B-cell lymphoma cell lines used in this study**

| **Cell line** | **Origin** | **FISH** | | | **BCL6 expression** |
|---|---|---|---|---|---|
| | | ***BCL2*** | ***MYC*** | ***BCL6*** | |
| **Ramos** | Burkitt lymphoma EBV negative | normal | rearrangement | gain | +++ |
| **DG75** | Burkitt lymphoma EBV negative | gain | rearrangement | normal | ++ |
| **Raji** | Burkitt lymphoma EBV positive | normal | rearrangement | normal | ++ |
| **Ly03** | ABC-DLBCL | amplification | gain | gain | + |
| **Toledo** | GC-DLBCL | gain and rearrangement | gain | normal | - |

**Supplementary Table S2. Cell proliferation analysis (WST-1 method) in (a) Ramos and (b) Raji cells using different concentrations of Romidepsin and JQ1 to generate Combination Index (CI) plot**

**a**

| Romid (nM) | JQ1 (µM) | Relative Metabolic Activity | Combination Index (CI) |
|---|---|---|---|
| 0 | 0 | 1.00 | |
| 1.0 | 0.1 | 0.94 | 1.32 |
| **5.0** | **1.0** | 0.26 | 0.73 |
| 10.0 | 1.0 | 0.27 | 1.16 |
| 2.0 | 0.5 | 0.55 | 0.76 |
| 5.0 | 0.5 | 0.27 | 0.59 |
| 10.0 | 0.5 | 0.28 | 1.03 |
| 15.0 | 1.5 | 0.27 | 1.77 |

**b**

| Romid (nM) | JQ1 (µM) | Relative Metabolic Activity | Combination Index (CI) |
|---|---|---|---|
| 0 | 0 | 1.00 | |
| 1.0 | 0.1 | 0.57 | 0.80 |
| **5.0** | **1.0** | 0.29 | 0.50 |
| 10.0 | 1.0 | 0.29 | 0.99 |
| 2.0 | 0.5 | 0.29 | 0.20 |
| 5.0 | 0.5 | 0.29 | 0.52 |
| 10.0 | 0.5 | 0.28 | 0.98 |
| 15.0 | 1.5 | 0.28 | 1.40 |

**Supplementary Table S3. Primers used for RT-PCR analysis**

| Name | Primers | Amplicon size |
|---|---|---|
| BCL6 | 5´-AGAGCCCATAAAACGGTCCT-3´<br>5´-AGTGTCCACAACATGCTCCA-3´ | 231 bp |
| CCND2 | 5´-ATATCCCGCACGTCTGTAGG-3´<br>5´-TGAGCTGCTGGCTAAGATCA-3´ | 176 bp |
| PRDM1 | 5´-CTGAGAGTGCACAGTGGAGA-3´<br>5´-TGGGTCTTGAGATTGCTGGT-3´ | 167 bp |
| PAX5 | 5´-AGACTTGTTCACACAGCAGCA-3´<br>5´-AGATTGGCCTTCATGTCGTC-3´ | 165 bp |
| XBP1 | 5´-GGAGTTAAGACAGCGCTTGG-3´<br>5´-GAGATGTTCTGGAGGGGTGA-3´ | 168 bp |
| RPS14 | 5´-TCACCGCCCTACACATCAAACT-3´<br>5´-CTGCGAGTGCTGTCAGAGG-3´ | 157 bp |

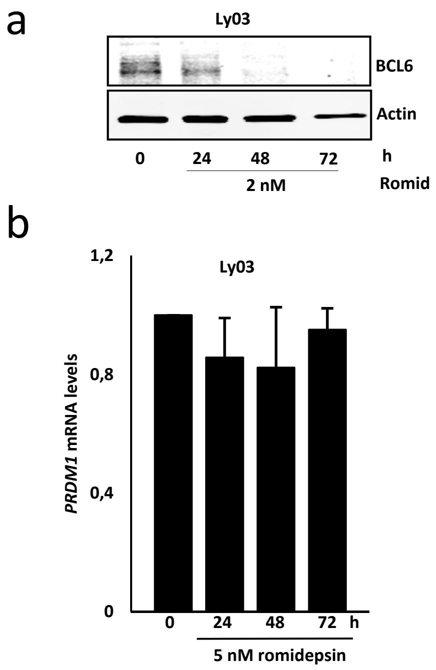


**Supplementary Figure S1. Romidepsin effects on BCL6 and PRDM1 expression in Ly03 cells.** (**a**) Western blot showing BCL6 downregulation in Ly03 cells treated with romidepsin for the indicated times. Actin was used as loading control. (**b**) RT-PCR showing PRDM1 mRNA expression in Ly03 cells treated with romidepsin as indicated.

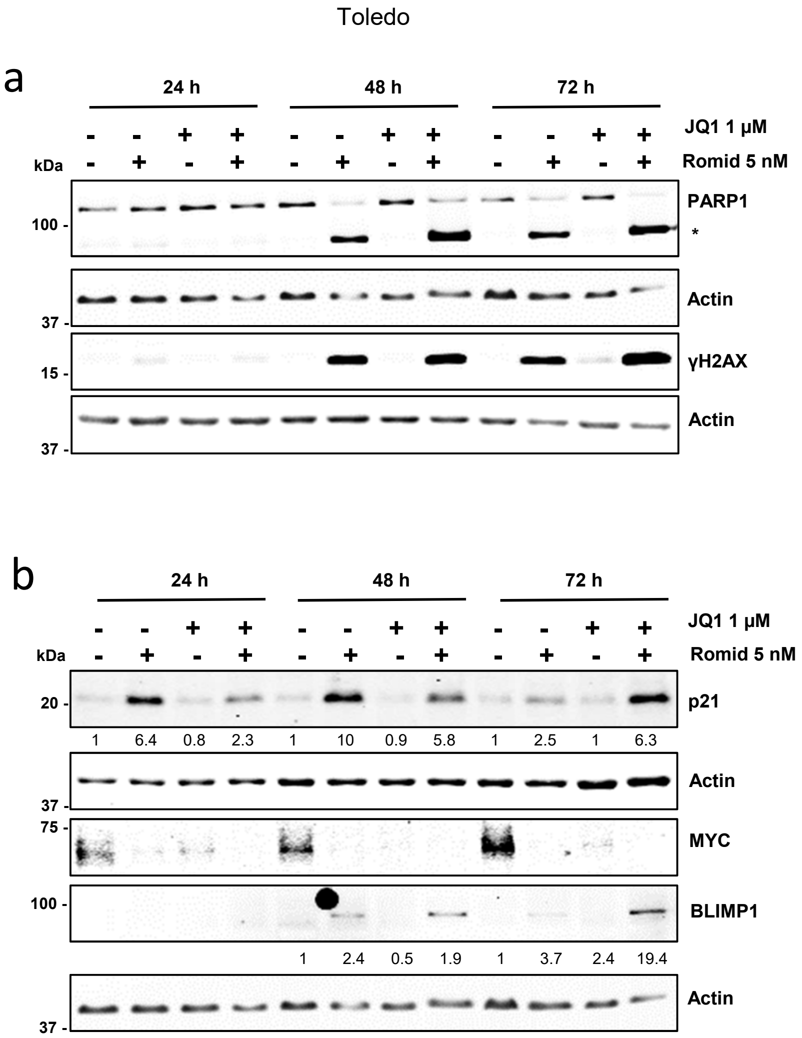


**Supplementary Figure S2. Synergistic effects of romidepsin and JQ1 in Toledo cells.** (**a**) Western blot showing cleaved PARP1 and γH2AX protein levels in Toledo cells treated with romidepsin and/or JQ1 for the indicated times. Actin was used as loading control. (**b**) Western blot showing p21, MYC and BLIMP1 protein levels in Toledo cells treated as above.

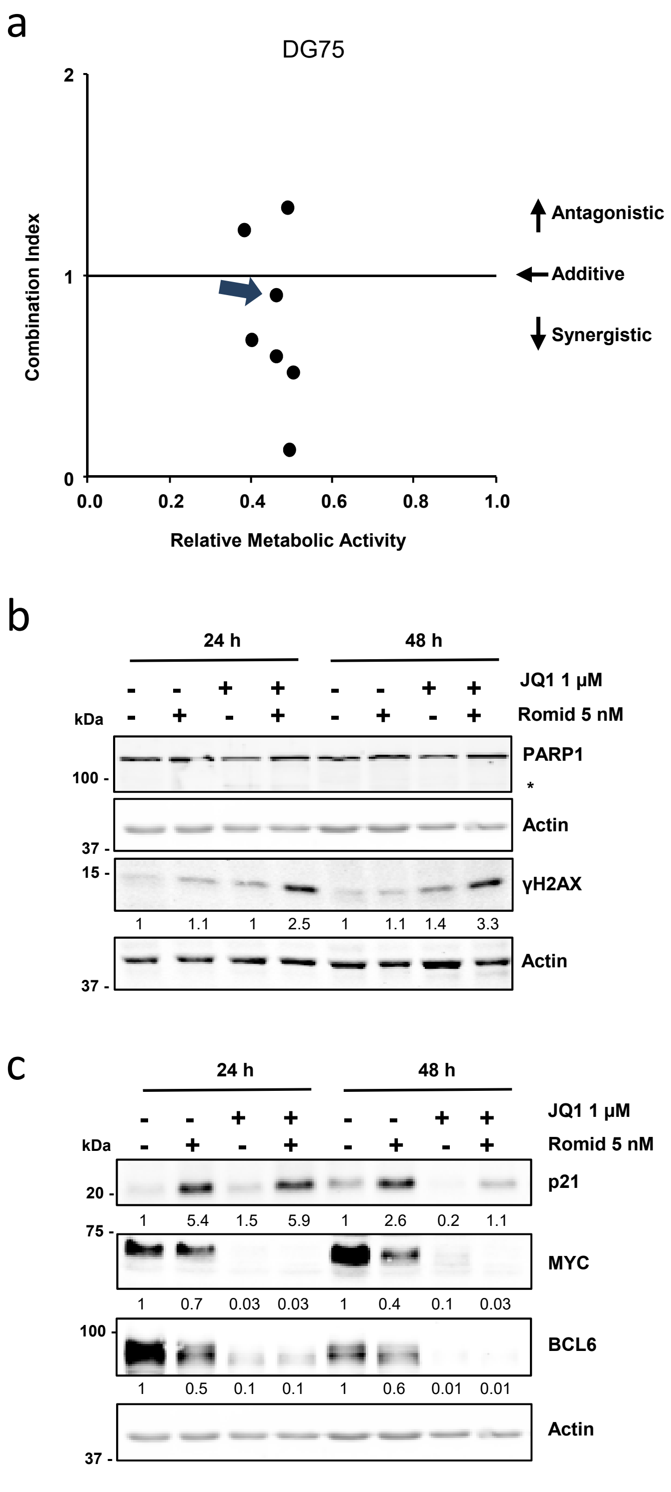


**Supplementary Figure S3. Synergistic effects of romidepsin and JQ1 in DG75 cells.** (**a**) Combination index plot showing synergistic effect of romidepsin plus JQ1 on the proliferation of DG75 cells. (**b**) Western blot showing PARP1 and γH2AX protein levels in DG75 cells treated with romidepsin and/or JQ1 for the indicated times. Actin was used as loading control. (**c**) Western blot showing p21, MYC and BCL6 protein levels in DG75 cells treated as above.

C

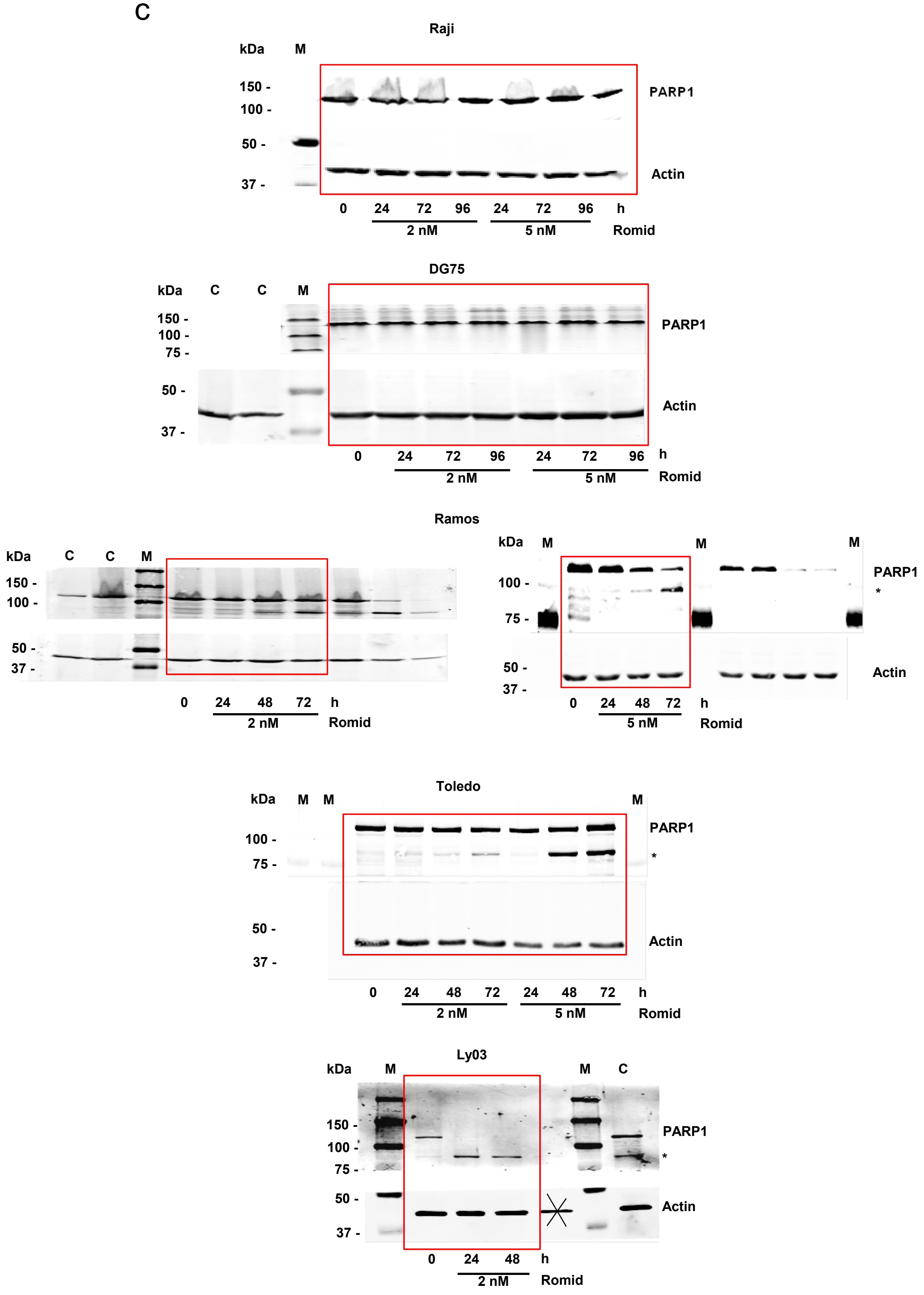


**Supplementary Figure S4.** Full-length blots corresponding to Figure 1c. (M, markers. C, positive control)

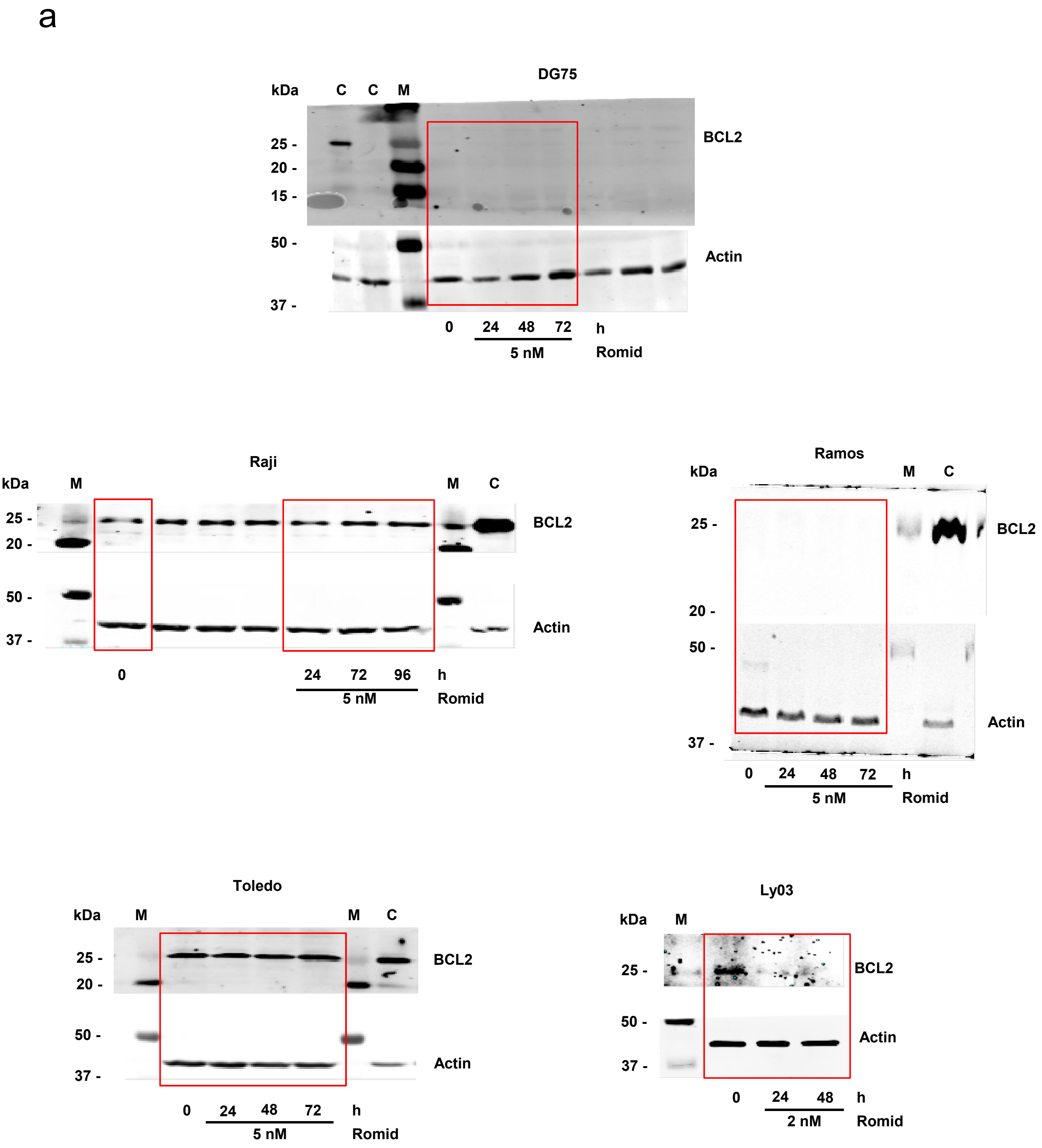


**Supplementary Figure S5.** Full-length blots corresponding to Figure 2a. (M, markers. C, positive control).

b

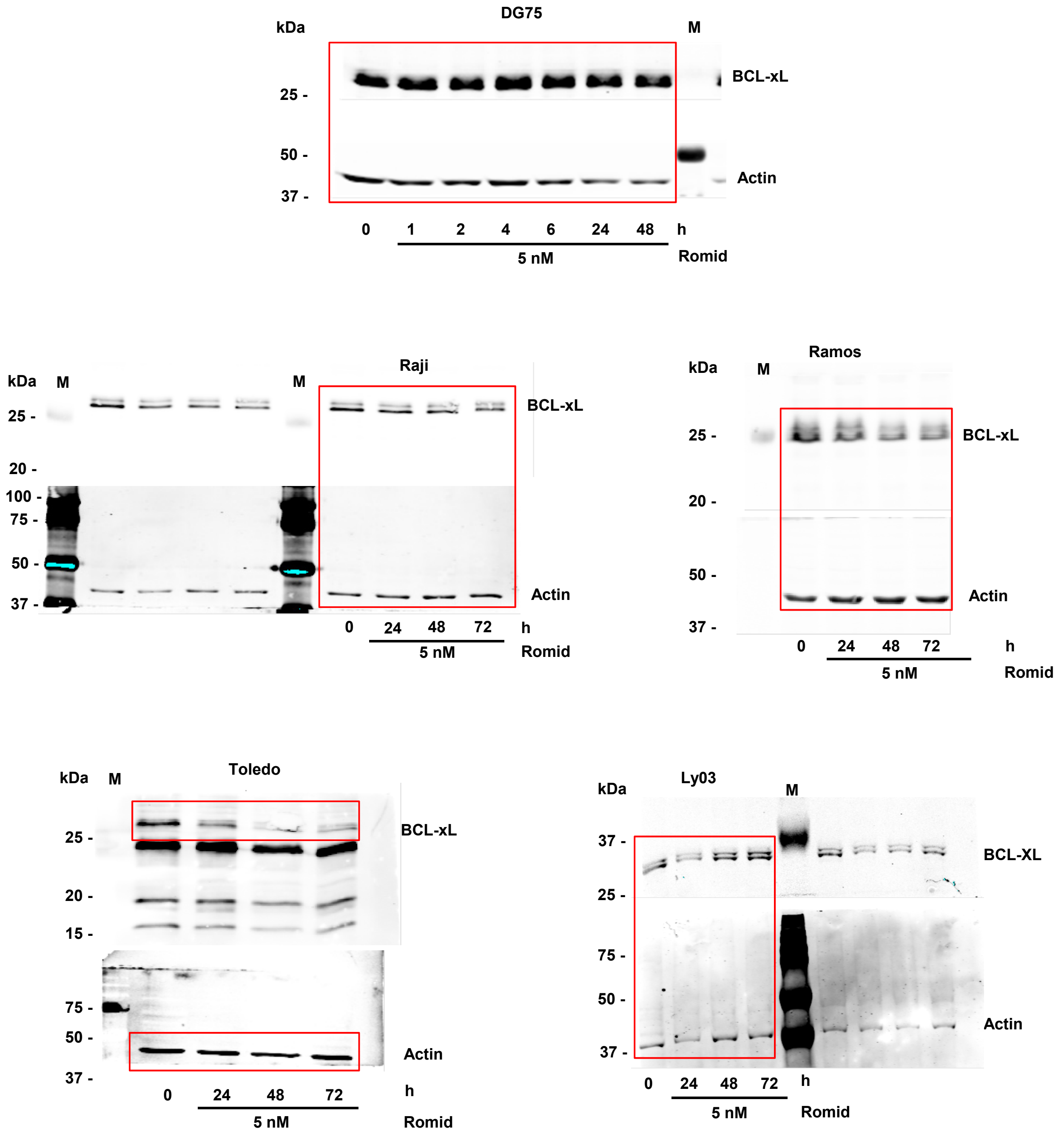


**Supplementary Figure S6.** Full-length blots corresponding to Figure 2b. (M, markers. C, positive control).

C

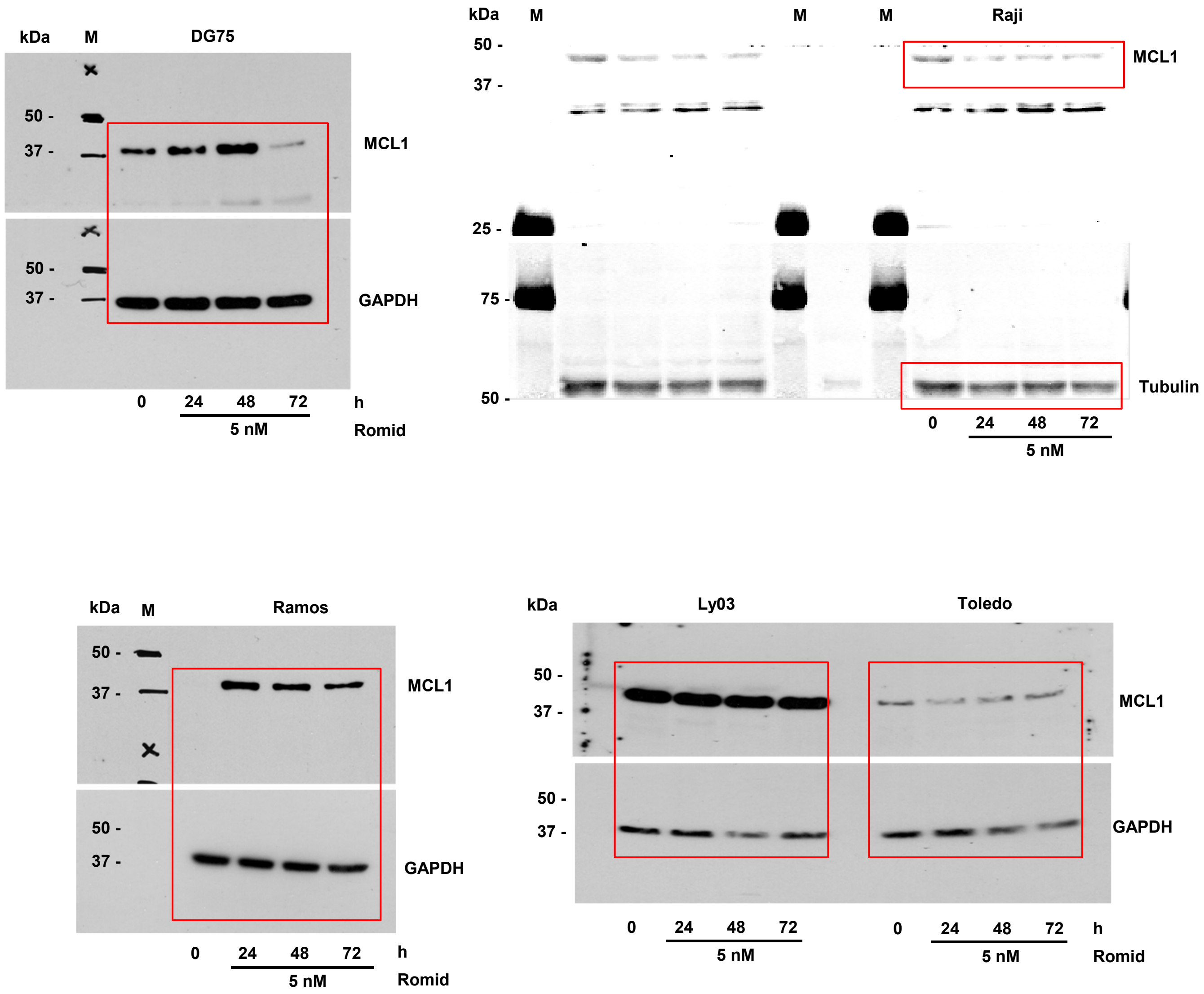


**Supplementary Figure S7.** Full-length blots corresponding to Figure 2c. (M, markers. C, positive control).

d

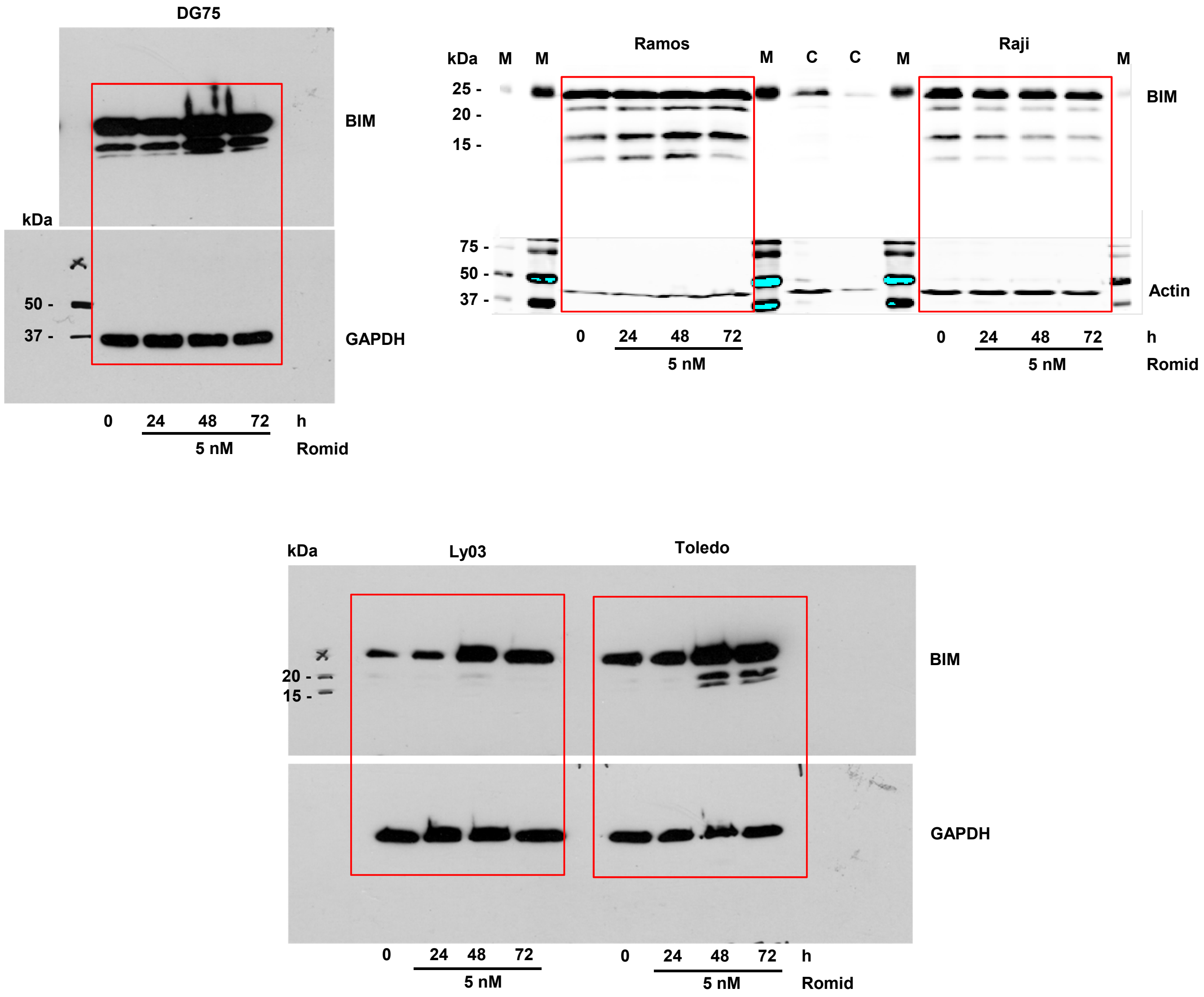


**Supplementary Figure S8.** Full-length blots corresponding to Figure 2d. (M, markers. C, positive control).

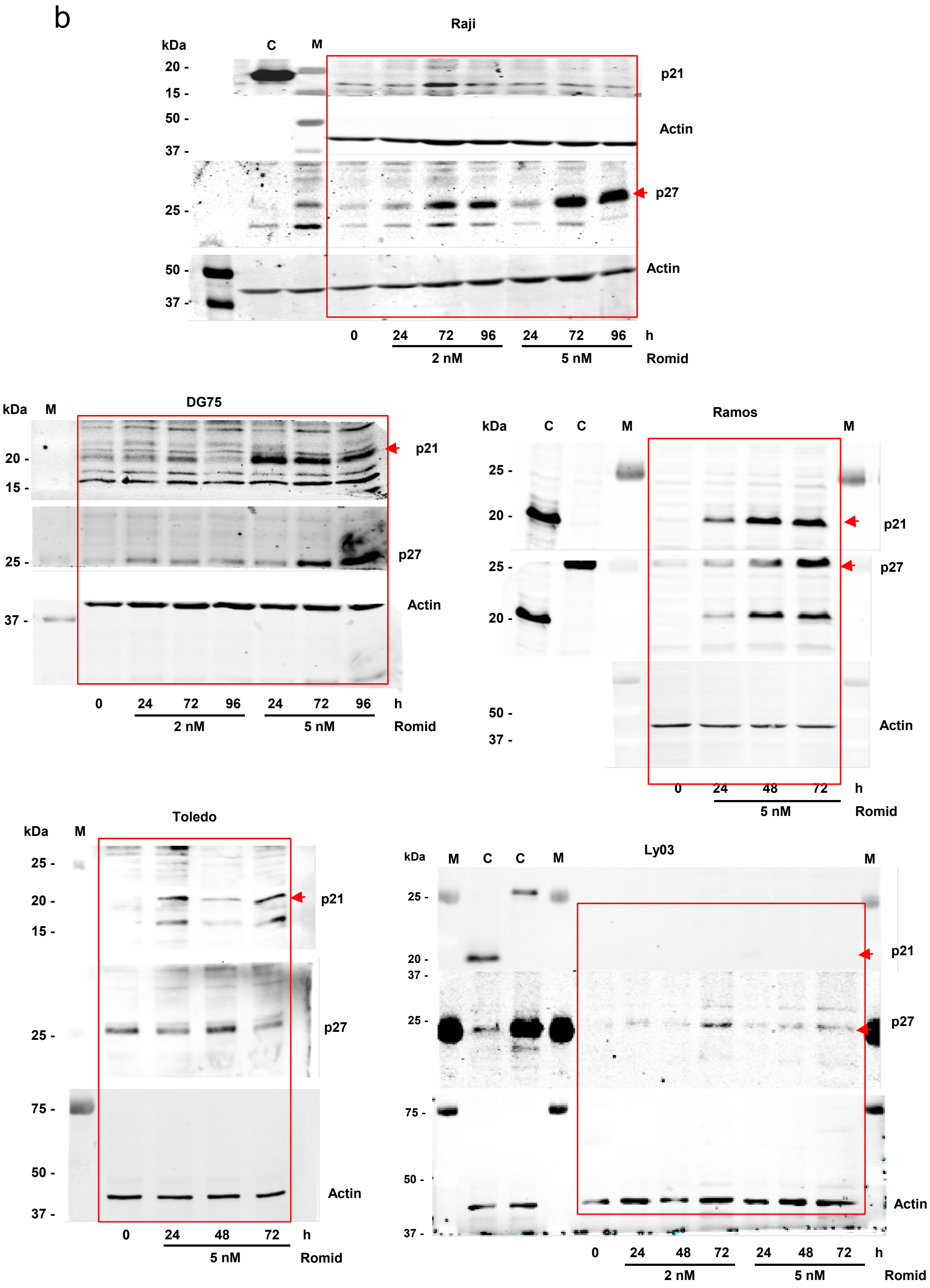


**Supplementary Figure S9.** Full-length blots corresponding to Figure 3. (M, markers. C, positive control)

a

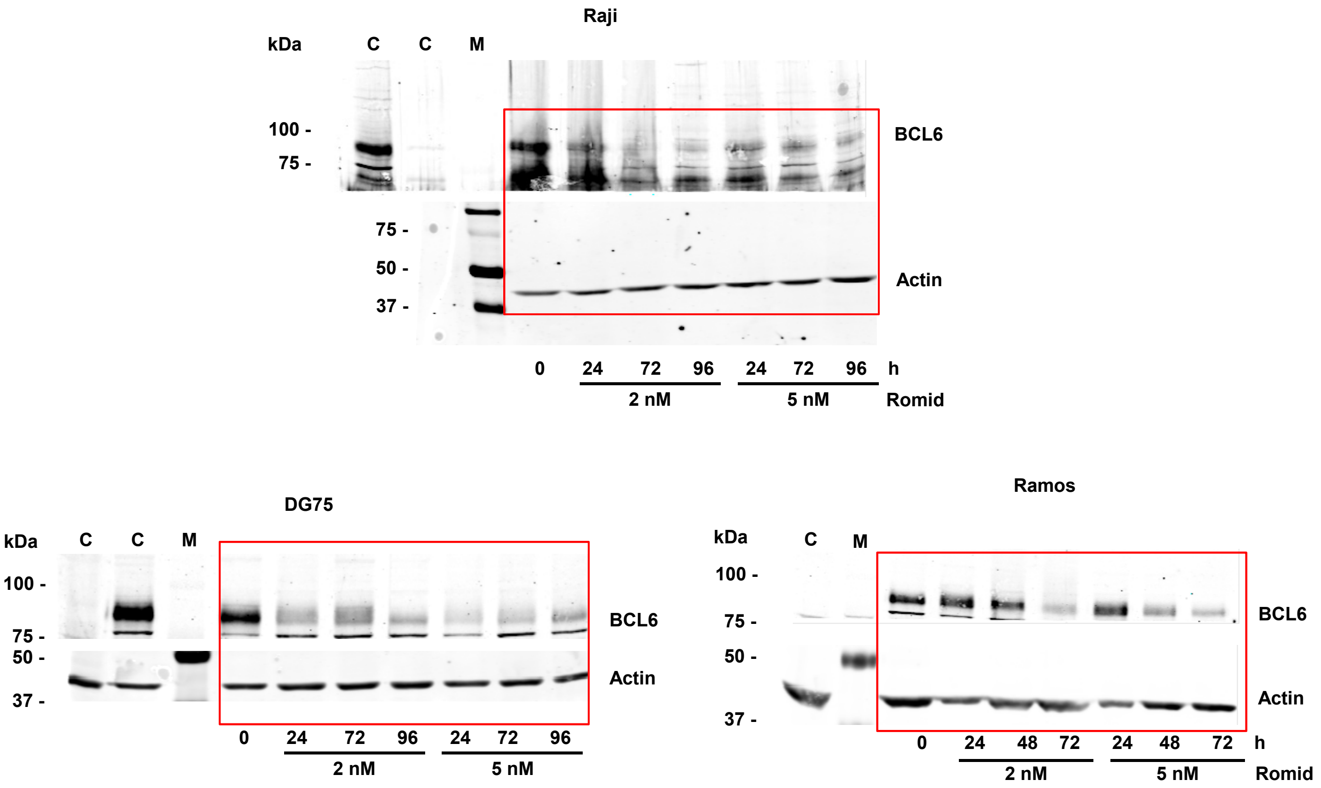


d

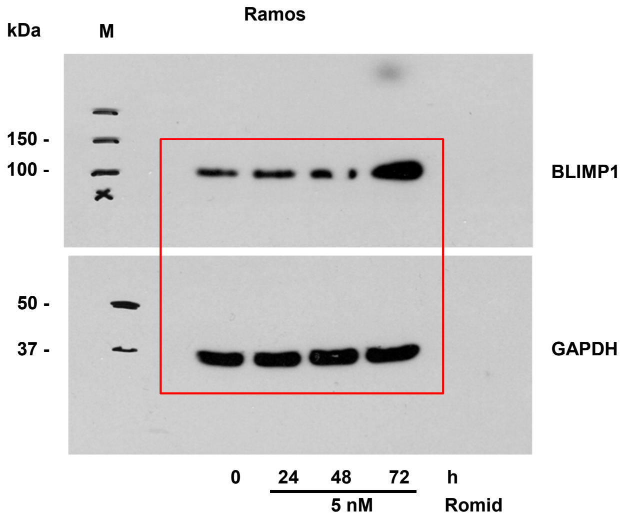


**Supplementary Figure S10.** Full-length blots corresponding to Figure 4. (M, markers. C, positive control).

a

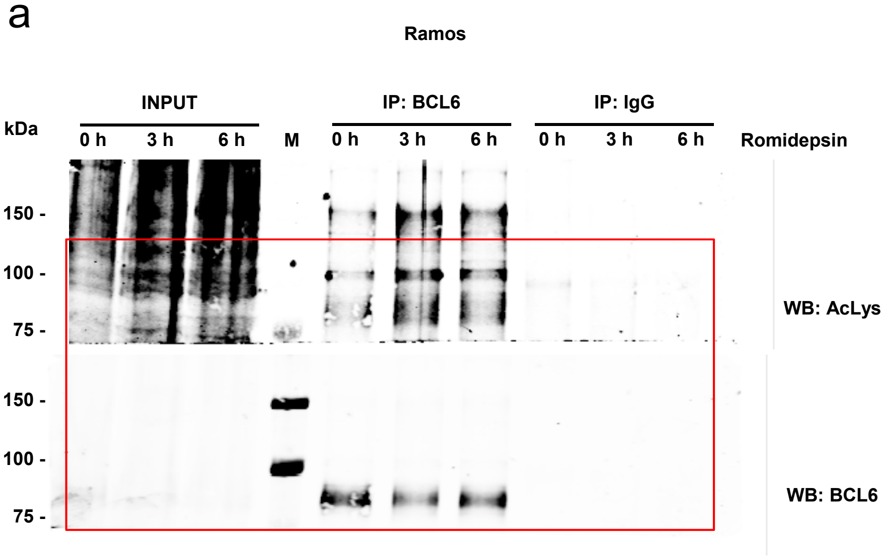


d

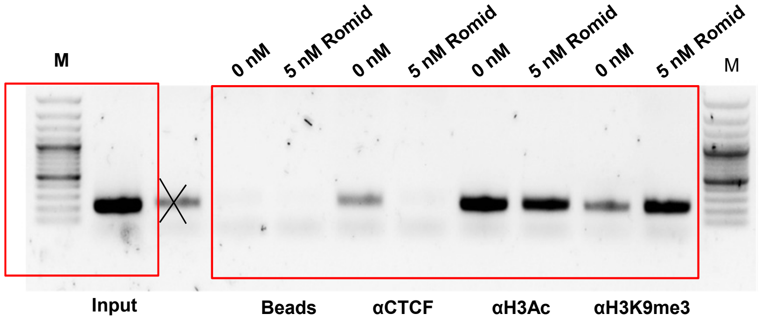


**Supplementary Figure S11.** Full-length blots corresponding to Figure 5. (M, markers).

d

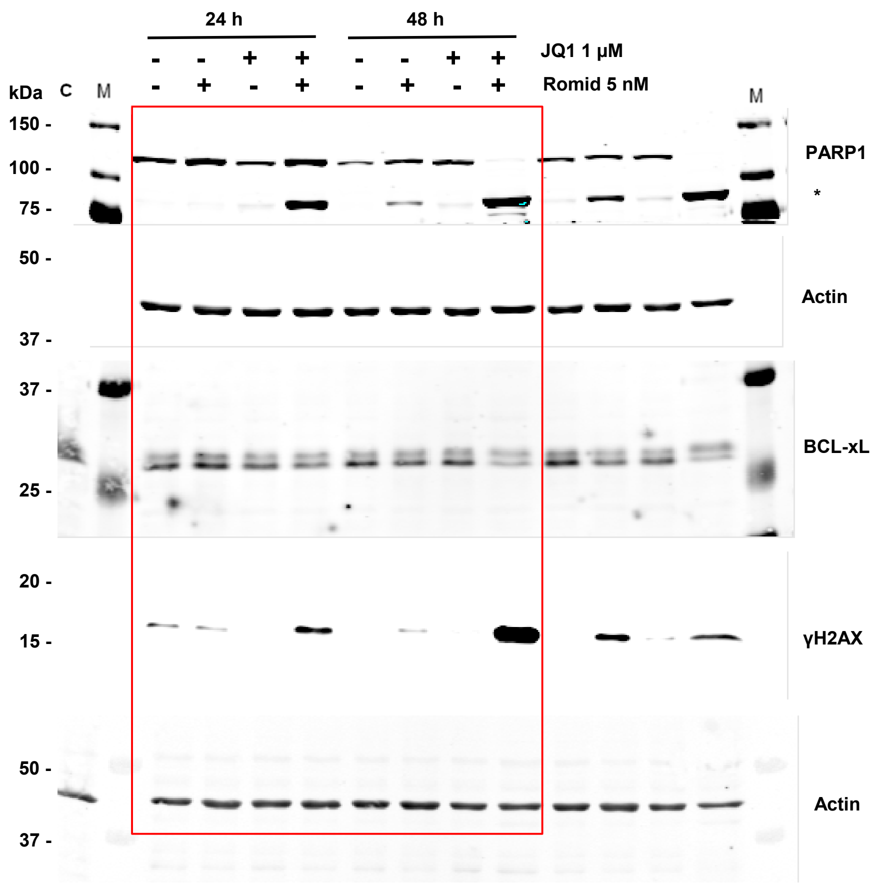


**Supplementary Figure S12.** Full-length blots corresponding to Figure 6d. (M, markers. C, positive control).

e

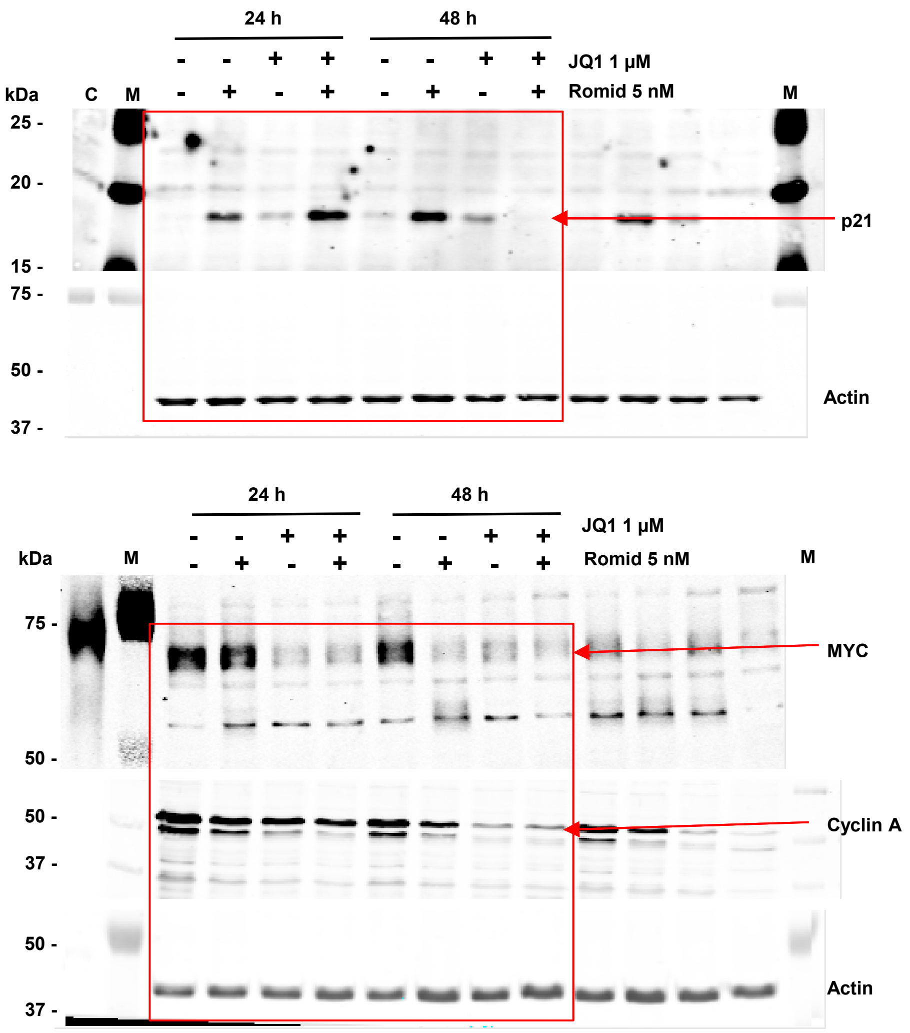


f

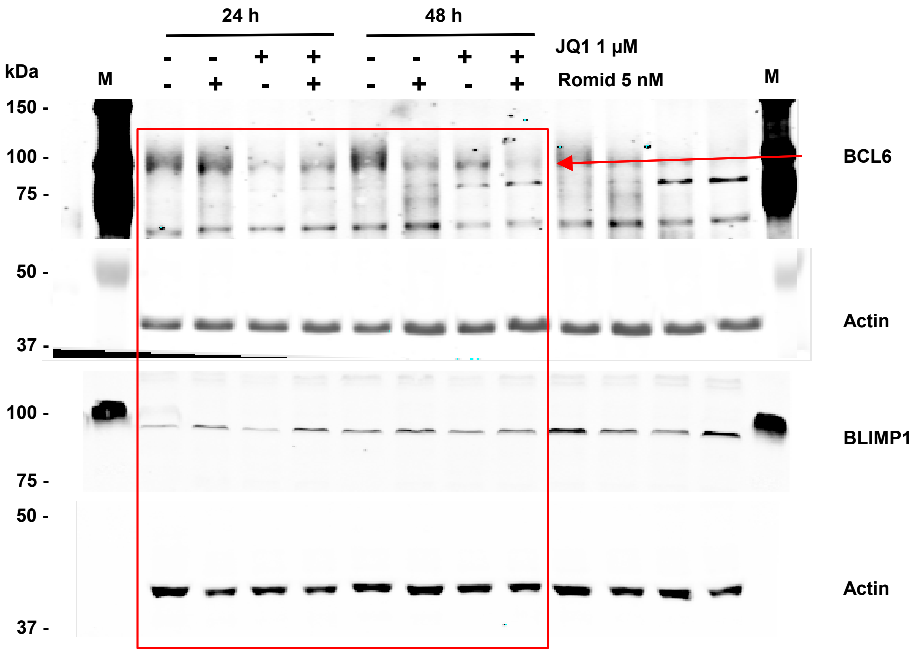


**Supplementary Figure S13.** Full-length blots corresponding to Figure 6e and f. (M, markers. C, positive control).

b

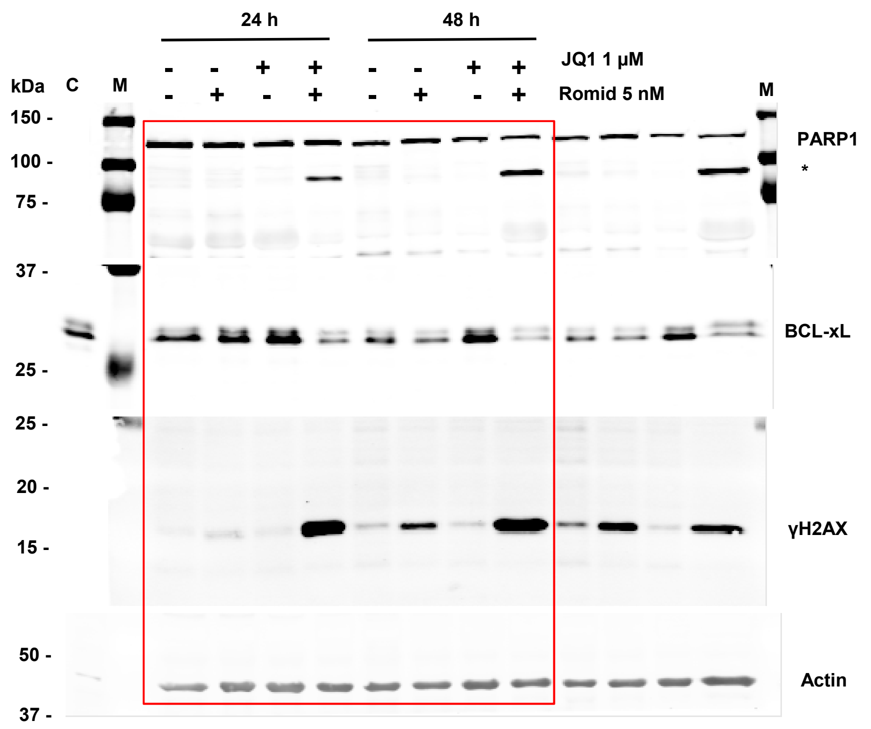


c

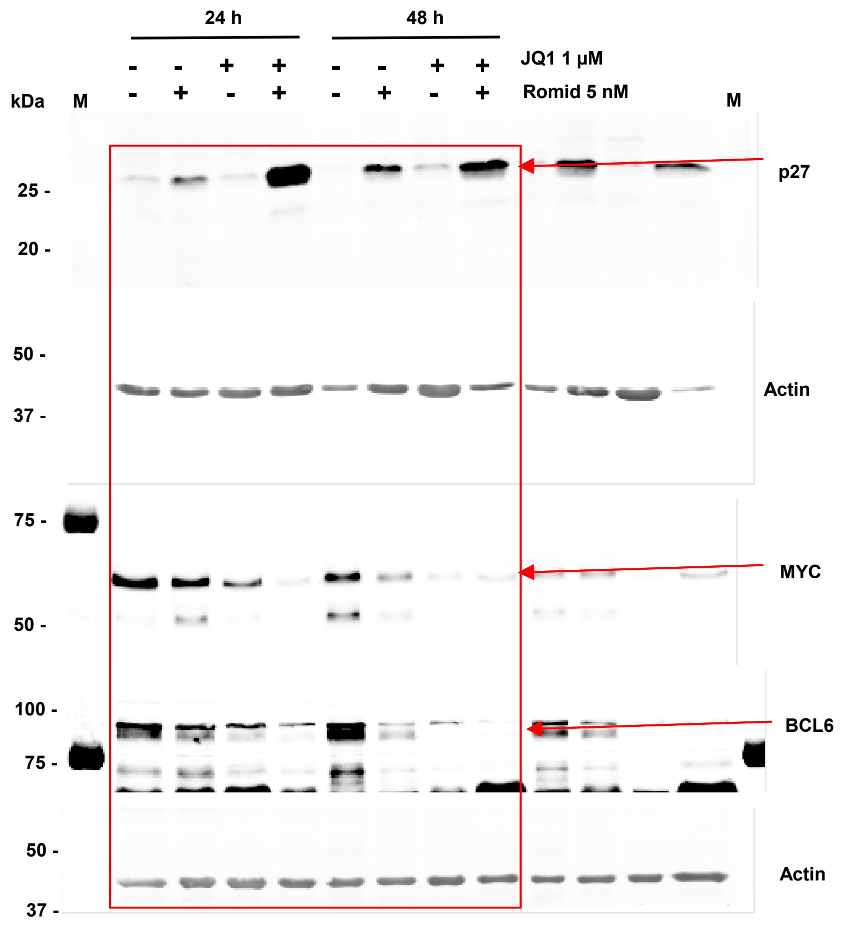


**Supplementary Figure S14.** Full-length blots corresponding to Figure 7 (M, markers. C, positive control).

a

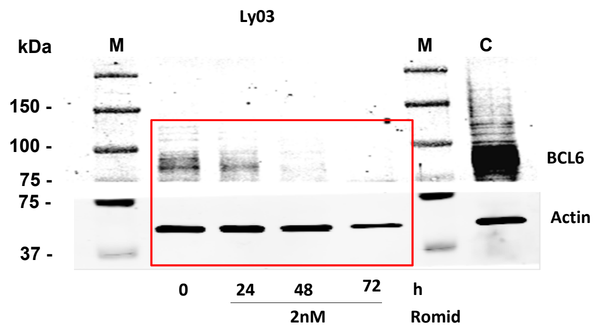


**Supplementary Figure S15.** Full-length blots corresponding to Figure S1a. (M, markers; C, positive control).

b

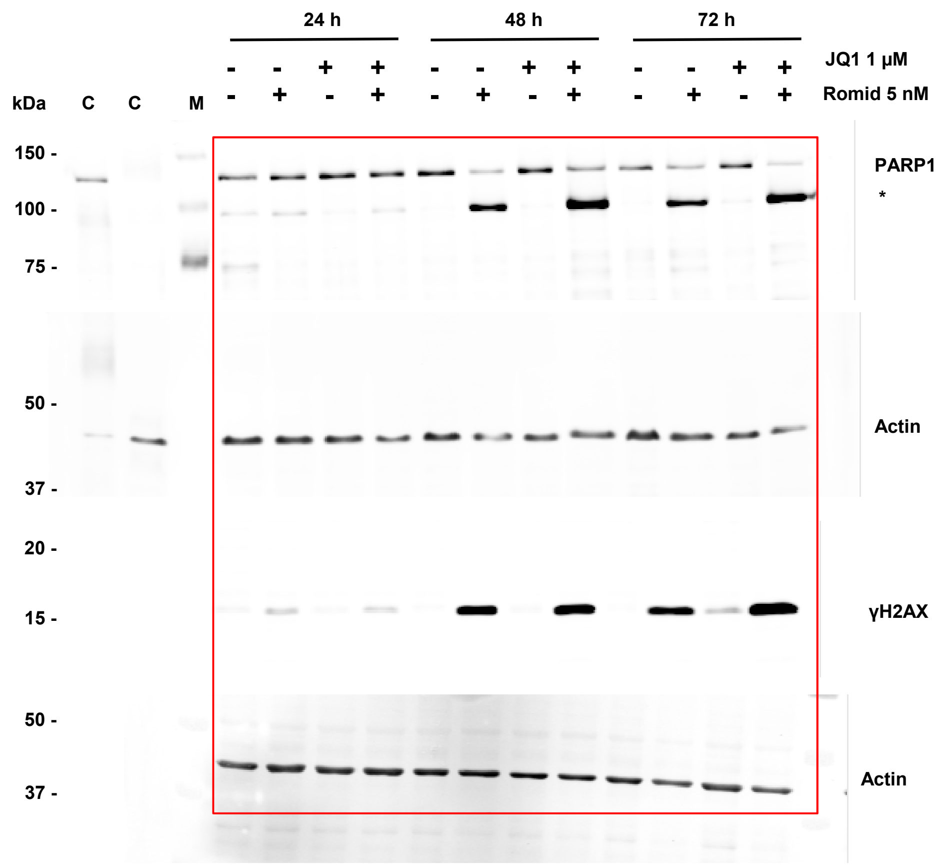


**Supplementary Figure S16.** Full-length blots corresponding to Figure S2b. (M, markers; C, positive control).

C

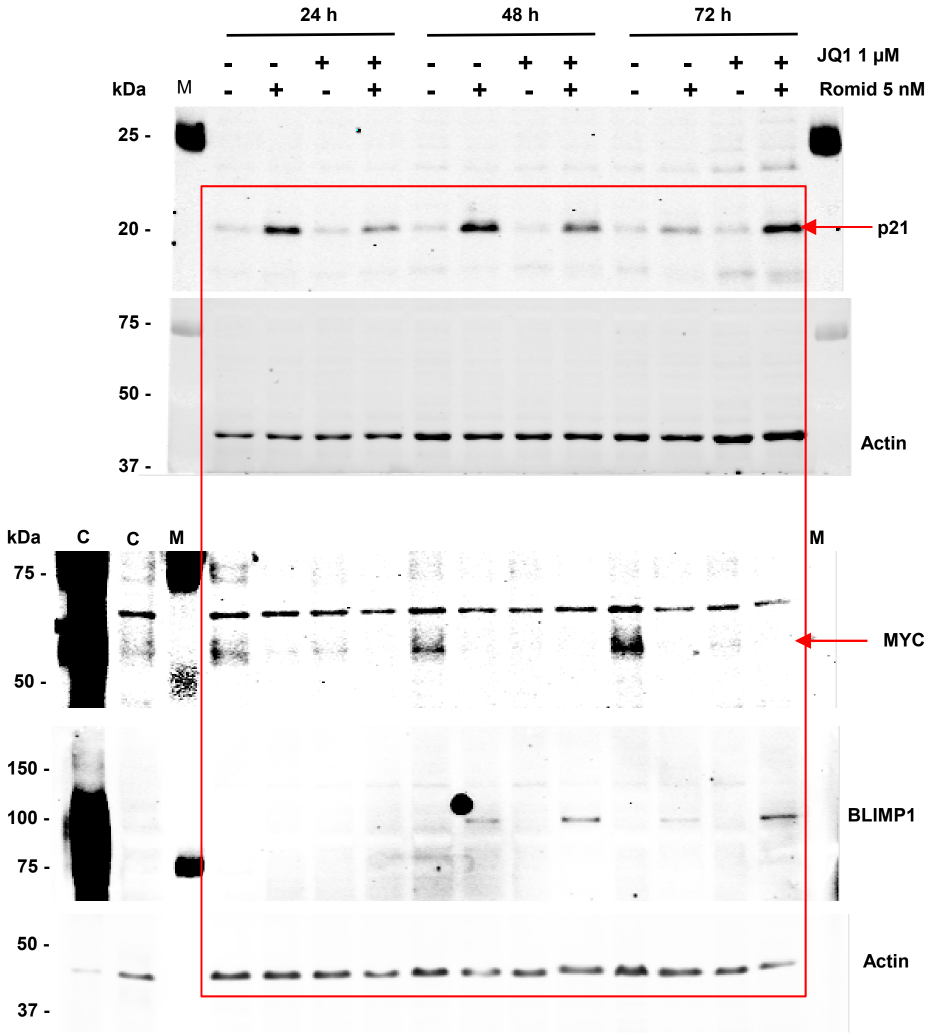


**Supplementary Figure S17.** Full-length blots corresponding to Figure S2c. (M, markers; C, positive control).

b

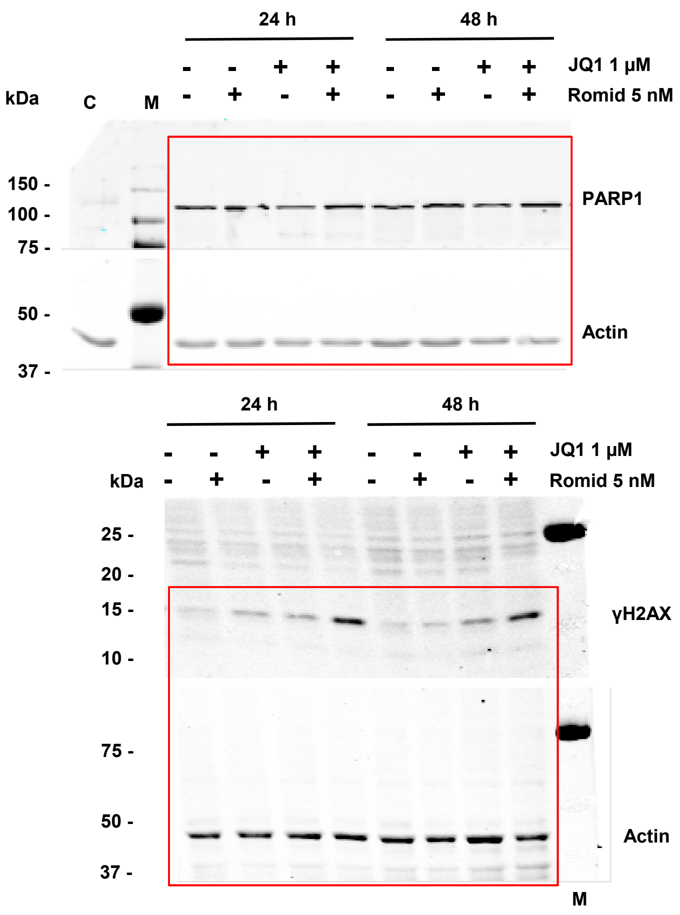


c

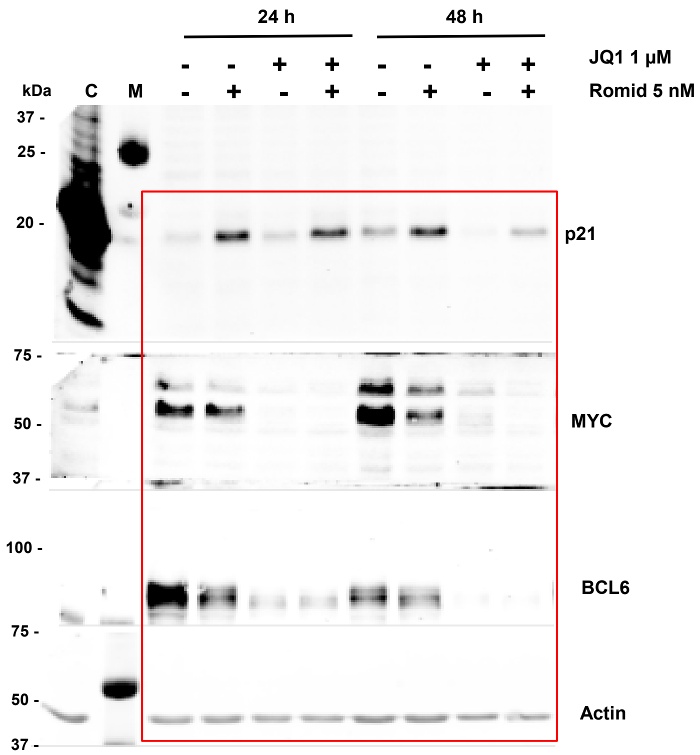


**Supplementary Figure S18.** Full-length blots corresponding to Figure S3. (M, markers; C, positive control).
